# Novel insights of acupuncture in ischemic stroke: orchestrating neuro-endocrine-immune network

**DOI:** 10.3389/fimmu.2026.1772371

**Published:** 2026-03-19

**Authors:** Xinyu Zhou, Zhongren Sun, Yang Cui, Hongna Yin

**Affiliations:** 1 Heilongjiang University of Chinese Medicine, Harbin, China; 2 The Second Affiliated Hospital of Heilongjiang University of Chinese Medicine, Harbin, China

**Keywords:** acupuncture, electroacupuncture, ischemic stroke, mechanism, neuro-endocrine-immune network

## Abstract

Ischemic stroke (IS) is a major public health challenge with high rates of mortality and disability. Its pathophysiology is complex and multifactorial. Although the pathogenesis of IS originates from local cerebral ischemia, it progresses to a systemic disorder involving dysfunction of the neuro-endocrine-immune (NEI) network. Acupuncture, a non-pharmaceutical therapy characterized by holistic regulation, holds considerable potential for correcting multi-system imbalances after IS. Nevertheless, how acupuncture coordinates this multi-system network remains poorly understood. This review synthesizes current evidence on the cellular and molecular mechanisms underlying acupuncture’s regulation of neural signaling, hormonal homeostasis, and inflammatory responses following IS. Notably, we clarify how acupuncture modulates the NEI network via the cholinergic anti-inflammatory pathway and the brain-gut axis, emphasizing its multi-system synergistic effects. In addition, we analyze the main challenges in this field and look forward to the prospects. This comprehensive perspective provides new insights into the mechanism of acupuncture for IS, signifying a transition from empirical treatment to precision medicine.

## Graphical Abstract

**Figure:**
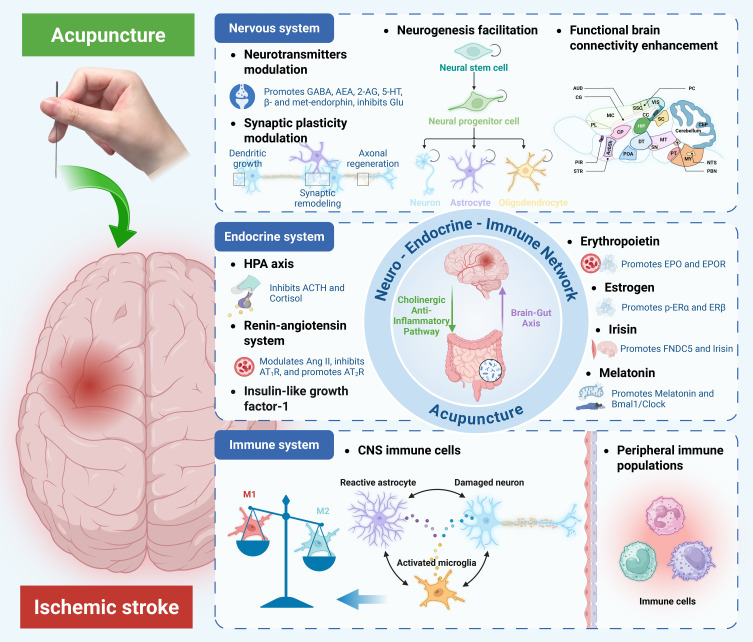
Created in BioRender. Zhou, X. (2026) https://BioRender.com/rndrzne.

## 1 Introduction

Stroke is the second leading cause of death and the third leading cause of disability worldwide, with approximately 17 million new cases each year (1, 2). According to the Global Stroke Fact Sheet 2025, the global stroke incidence rate has shown a marked deceleration, and worrying signs of reversal have emerged in low- and middle-income countries (3). The total global cost related to stroke is estimated to exceed $891 billion, accounting for roughly 1.12% of the world’s GDP (4). Ischemic stroke (IS) is the most common type, representing approximately 60-70% of all stroke cases (1). As population aging accelerates, the burden of IS is expected to increase further, placing significant strain on healthcare systems worldwide (5).

Growing evidence indicates that IS extends beyond intracranial pathology and becomes a systemic disease that disrupts network homeostasis (6, 7). After the onset of IS, the rapid activation of the autonomic nervous system and the hypothalamic-pituitary-adrenal axis (HPA axis) trigger the release of large quantities of hormones, facilitating the entry of immune cells and intestinal microbiota-derived components into the circulation (8, 9). Meanwhile, the damage-associated molecular patterns released by dead cells enter the blood circulation, activating peripheral immune cells and triggering a systemic inflammatory response (10). Subsequently, these components can feed back to the central nervous system (CNS), thereby worsening neuroinflammation throughout the brain and overactivating the neuroendocrine system (11, 12). Notably, this persistent crosstalk can lead to splenic atrophy, lymphocyte apoptosis, bone marrow dysfunction and intestinal ecological dysbiosis, forming a harmful feedback loop that exemplifies the breakdown of neuro-endocrine-immune (NEI) network homeostasis (13).

The NEI network is an integrated system where neural, endocrine, and immune components function as a coordinated whole through bidirectional communication, rather than as separate entities (14). This differs from parallel multi-system effects, which occur independently without mutual interaction. Genuine NEI network coordination is defined by functional interdependence—when one component is perturbed, the others respond in compensatory or synergistic ways (15). Such interactions create emergent properties (1 + 1 + 1>3) that cannot be predicted from any single system alone. Based on this perspective, we propose that IS-induced brain dysfunction may initiate and amplify pathological NEI cascades, establishing a vicious cycle that worsens neural damage and systemic decline. Therefore, targeting the NEI network represents a highly promising therapeutic strategy.

Acupuncture has been incorporated into the clinical management guidelines of IS in many countries (16). Simultaneously, experimental studies have gradually clarified the modern biological mechanisms of acupuncture treatment for IS (17–19). The reported mechanisms include neurotransmitter regulation, neural plasticity, brain functional connectivity, endocrine modulation and immune inflammatory responses. Notably, these multi-dimensional effects are highly consistent with the feature of NEI network mechanism, indicating that acupuncture may improve IS by coordinating this network (20–23). Nevertheless, there is still a huge knowledge gap in this field.

Consequently, this review synthesizes the current evidence (**Supplementary Table 1** summarizes the characteristics of the included studies), clarifies the cellular and molecular mechanisms by which acupuncture regulates various components of the NEI network, and emphasizes how acupuncture coordinates multi-system interactions to combat IS. This comprehensive perspective not only offers novel insights into the role of acupuncture in treating IS but also provides a scientific basis for advancing acupuncture from empirical practice to mechanism-based precision medicine.

## 2 Mechanisms of acupuncture on neuromodulation

Acupuncture exerts its therapeutic effects in IS primarily through multi-level neuromodulation. This section examines how acupuncture regulates neural homeostasis by targeting neurotransmitter systems, synaptic plasticity, neurogenesis, and functional brain networks.

### 2.1 Neurotransmitters modulation

Neural homeostasis in the CNS depends on a dynamic excitation-inhibition balance, primarily governed by bidirectional regulation of glutamate (Glu) and gamma-aminobutyric acid (GABA), and finely modulated by multi-level networks such as the endocannabinoid system and the monoamine neurotransmitters (24, 25). IS disrupts this excitation-inhibition equilibrium, triggering a cascade of pathological events: Glu hyperactivity induces excitotoxicity, impaired GABA inhibition exacerbates neuronal hyperexcitability, and endocannabinoid dysregulation undermines neuroprotective function (26–28). Acupuncture rebalances the Glu/GABA metabolism in middle cerebral artery occlusion (MCAO) rats, contributing to neuroprotection (29, 30).

#### 2.1.1 Glutamate

Glu is the main excitatory neurotransmitter in the CNS. It transmits signals between synapses through ionotropic Glu receptors (mainly α-amino-3-hydroxy-5-methyl-4-isoxazolepropionic acid receptors and N-methyl-D-aspartate receptors) and metabotropic Glu receptors (31). In ischemic conditions, abnormal release and reuptake of Glu lead to its pathological accumulation in the synaptic cleft, resulting in prolonged receptor overactivation, intracellular Ca^2+^ and Na^+^ overload, and ultimately neuronal death (32). Acupuncture can lower cerebral Glu levels in ischemia-reperfusion models, alter the expression of N-methyl-D-aspartate receptor subunit 2A and 2B and the transcription of metabotropic receptor 1a, lessen Ca^2+^ influx and excitotoxicity, and improve neurological deficits (30, 33, 34). Additionally, electroacupuncture (EA) pretreatment inhibits the activation of the N-methyl-D-aspartate receptor subunit 2B/muscle calpain/p38 mitogen-activated protein kinase (p38) pathway in the hippocampal CA1 region. This process prevents neuronal apoptosis and improves functional recovery (35). The Glu transporter 1, which is specifically expressed on astrocytes, mediates over 90% of Glu reuptake and is essential for maintaining extracellular Glu homeostasis (36, 37). EA can alleviate the excessive activation of astrocytes, upregulate the expression of Glu transporter 1, and reduce the extracellular Glu concentration, which improves the neurological prognosis (38, 39).

#### 2.1.2 Gamma-aminobutyric acid

GABA is the main neurotransmitter in the CNS responsible for inhibitory synaptic transmission (40). It is synthesized from Glu by glutamic acid decarboxylase and degraded by GABA transaminase (41). Under physiological conditions, GABAergic neurons release GABA, which acts on GABA_A_ and GABA_B_ receptors (GABA_A_R and GABA_B_R) on the postsynaptic membrane. This process enables Cl^−^ to enter the cells and generates inhibitory potentials. Moreover, K^+^-Cl^−^ cotransporter 2 and Na^+^-K^+^-2Cl^−^ cotransporter 1 actively transport ions to maintain a low intracellular Cl^−^concentration, ensuring the efficacy of inhibitory neurotransmission (42). Under pathologic conditions, the GABAergic system is impaired, as evidenced by reduced GABA levels, decreased receptor expression, and impaired K^+^-Cl^−^ cotransporter 2 function. These changes cause the accumulation of Cl^−^ within cells, synaptic depolarization, and aberrant neuronal discharges (43–45). Acupuncture pretreatment inhibits the decline in the action potential firing of cortical GABAergic neurons and prevents synaptic transmission dysfunction caused by ischemia, maintaining the electrophysiological homeostasis of GABAergic neurons (46). In response to decreased GABA levels, acupuncture upregulates the GABA levels and the expression of GABA_A_Rγ2 and GABA_B_R in the injured brain tissue, laying the foundation for restoring inhibitory neural transmission (47, 48). Further study demonstrated that EA regulates the GABA_B_R/cyclic adenosine monophosphate/protein kinase A/cAMP response element binding protein signaling pathway, promoting motor recovery (49). Acupuncture also boosts the production and release of GABA in motor neurons, strengthens the K^+^-Cl^−^ cotransporter 2-dependent control of GABAergic signaling, inhibits GABA transaminase activity, and reduces α-motor neuron hyperexcitability. These effects collectively reduce hyperactive spinal H-reflexes and improve motor function (50–53).

#### 2.1.3 Endocannabinoid

The endocannabinoid system consists of two principal lipid mediators: anandamide involved in emotional regulation, and 2−arachidonoylglycerol highly abundant in the CNS (54). Acupuncture involves multiple levels of endogenous cannabinoid signaling to trigger neuroprotection (55). EA pretreatment increases the levels of anandamide and 2−arachidonoylglycerol in the ischemic penumbra region (56). This process activates neuronal cannabinoid receptor type 1, promotes the phosphorylation of extracellular signal-regulated kinase 1/2 (ERK1/2), glycogen synthase kinase-3β (GSK-3β) at Ser9, and signal transducer and activator of transcription 3 (STAT3) at Ser727, and facilitates the membrane translocation of protein kinase C epsilon, collectively mediating anti-apoptotic effects (57–60). In addition, EA triggers cannabinoid receptor type 1-dependent peroxisome proliferator-activated receptor gamma coactivator 1-alpha-mediated mitochondrial biogenesis, upregulates nuclear respiratory factor 1 and mitochondrial transcription factor A, and enhances mitochondrial DNA replication. Ultimately, this pathway improves mitochondrial ultrastructure and oxidative metabolism, reduces the release of cytochrome c, and thereby alleviates reperfusion injury (61, 62).

#### 2.1.4 Adenosine

Adenosine exerts its effects by binding to specific receptors in the nervous and immune systems and is an endogenous neuroprotective agent (63, 64). Among the four identified receptor subtypes, which have different biological functions, the adenosine A_1_ receptor plays a major role in neuroprotection (65). However, although extracellular adenosine significantly increases during ischemia, its therapeutic application in the CNS is limited by its short half-life and poor permeability across the blood-brain barrier (66). A single EA pretreatment augments the phosphorylation of GSK-3β through adenosine A_1_ receptor, consequently preventing hippocampal neuron apoptosis and ameliorating neurological deficits following cerebral ischemia (67–70).

#### 2.1.5 Dopamine, opioid peptides, and 5-hydroxytryptamine

Dopamine, a catecholamine neurotransmitter, plays a key role in reward pathways and neuroplasticity regulation (71). EA increases the expression of growth-associated protein 43 via dopamine receptors D2, thus improving neural plasticity (72). Opioid peptides are neurotransmitters secreted by structures such as the pituitary and exert their physiological effects primarily through four opioid receptor subtypes (73). β-endorphin and methionine-enkephalin are released upon acupuncture stimulation and then bind to δ- and μ- opioid receptors, exerting neuroprotective effects (48, 74). Additionally, EA increases the levels of 5-hydroxytryptamine (5-HT) and its transporter SERT while inhibiting excessive activation of the 5-HT receptor 2A, thereby improving neurological function after IS (75).

**Figure 1:**
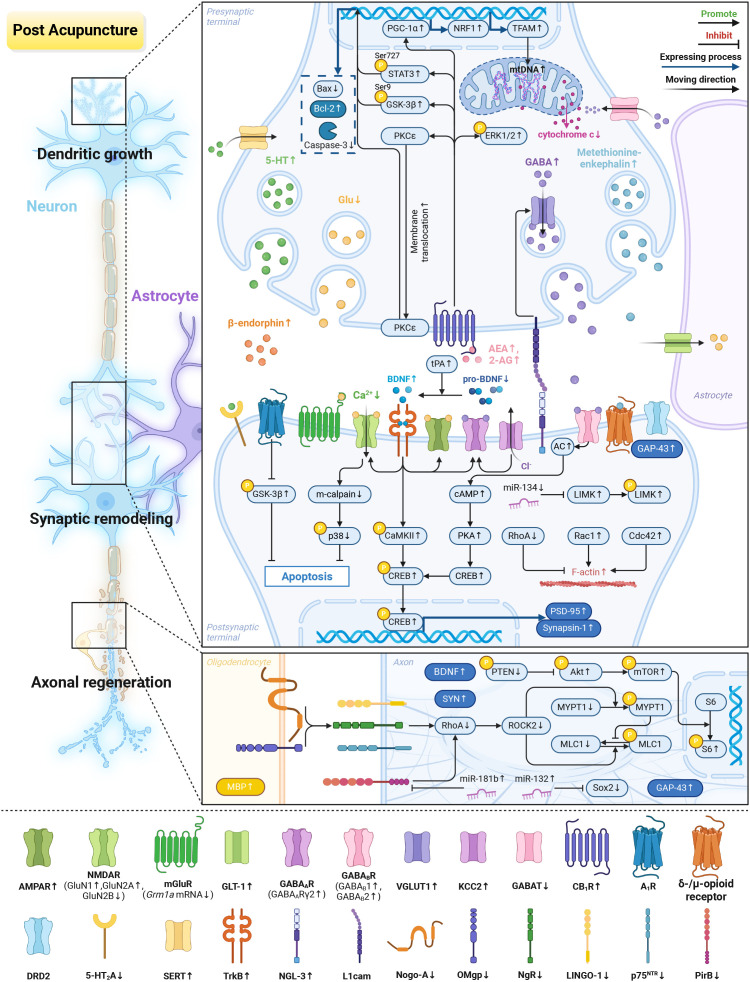
Neurotransmitters and synaptic plasticity modulation effects of acupuncture. **(A)** Neurotransmitters modulation: (1) Glu: acupuncture reduces Glu levels, modulates the expression of receptor subunits (GluN2A, GluN2B, and Grm1a) and GLT-1, and decreases Ca^2+^ influx, which alleviates excitotoxicity. It suppresses the GluN2B/m-calpain/p38 pathway to inhibit apoptosis. (2) GABA: acupuncture increases GABA levels, upregulates its receptors (GABA_A_Rγ2 and GABA_B_R) and KCC2, and reduces GABAT expression. It activates the cAMP/PKA/CREB pathway via GABA_B_R, leading to the restoration of inhibitory neurotransmission. (3) Endocannabinoids: acupuncture increases AEA and 2-AG levels, which upregulates CB_1_R and its downstream ERK1/2, GSK-3β, STAT3 and PKCϵ signaling. These effects exert neuroprotective and anti-apoptotic effects. Acupuncture also stimulates the PGC-1α/NRF1/TFAM mitochondrial biogenesis pathway. (4) Adenosine and other monoamine neurotransmitters: acupuncture increases GSK-3β phosphorylation via A_1_R, which in turn inhibits the apoptotic process. It also upregulates GAP-43 through DRD2. Acupuncture induces the release of β-endorphin and methionine-enkephalin, which bind to δ-/μ-opioid receptors. Additionally, it promotes 5-HT secretion and SERT expression while suppressing 5-HT_2_A. **(B)** Synaptic plasticity modulation: (1) Synaptic reconfiguration: acupuncture increases the expression of AMPAR, GluN1 and GABA_A_R, CaMKII, PSD-95, and Synapsin-1 by activating the BDNF/TrkB pathway, which establishes the structural and functional basis for neural repair. Moreover, it activates the NGL-3/L1cam signal to improve VGLUT1 expression and excitatory synapse density. (2) Dendritogenesis: acupuncture modulates Rho GTPases (RhoA, Rac1, Cdc42) to promote F-actin polymerization. It decreases miR-134 to increase LIMK1, which drives dendritic spine growth. (3) Axonal repair: acupuncture downregulates Nogo-A, OMgp and the triple complex (NgR, p75^NTR^, and LINGO-1), while inhibiting the RhoA/ROCK pathway and downstream factors MYPT1 and MLC1, promoting growth cone formation and axon extension. Acupuncture upregulates miR-132, inhibits the Sox2 axis, and induces axon growth. It stimulates the Akt/mTOR/S6 pathway and upregulates miR-181b to inhibit PirB expression, promoting axon rewiring. In addition, acupuncture increases MBP expression to alleviate myelin sheath injury. Created in BioRender. Zhou, X. (2026) https://BioRender.com/n7ncds5.

In summary, acupuncture coordinates a multi-target neural regulatory network, fine-tunes the excitation-inhibition balance (through Glu and GABA), and engages multiple signaling pathways (such as endocannabinoids and adenosine) to restore neural homeostasis after IS (**Table 1**; **Figure 1**). Notably, acetylcholine (ACh) also represents a potential mediator of the needling effect. It is produced from choline and acetyl-CoA by the enzyme choline acetyltransferase (ChAT) (105). Given its role in the cholinergic anti-inflammatory pathway, this mechanism will be further detailed in the integrated mechanism section.

**Table 1:** Mechanisms of acupuncture in regulating the nervous system in ischemic stroke.

Category	Core molecules/processes	Primary functions	Key mechanisms	Refs
Neurotransmitters	Glu	Inhibits excitotoxicity	Glu ↓, GluN2A ↑, GluN2B ↓, Grm1a ↓, GLT-1 ↑	(30, 33, 34, 38, 39)
		Exerts anti-apoptotic effects	GluN2B/m-calpain/p38 signaling pathway ↓	(35)
	GABA	Restores inhibitory neural transmission	GABA ↑, GABA_A_Rγ2 ↑, KCC2 ↑, GABAT ↓	(47, 48, 50–53)
		Exerts neuroprotective effects	GABA_B_R/cAMP/PKA/CREB signaling pathway ↑	(49)
	Endocannabinoid	Exerts neuroprotective effects	AEA ↑, 2-AG ↑, CB_1_R/ERK1/2 signaling pathway ↑	(56, 57)
		Exerts anti-apoptotic effects	CB_1_R/GSK-3β signaling pathway ↑, CB_1_R/STAT3 signaling pathway ↑, CB_1_R/PKCϵ signaling pathway ↑	(58–60)
		Triggers mitochondrial biogenesis	CB_1_R/PGC-1α signaling pathway ↑	(61, 62)
	Adenosine	Exerts anti-apoptotic effects	GSK-3β ↑ (via A_1_R)	(67–70)
	Dopamine	Improves neural plasticity	GAP-43 ↑ (via DRD2)	(72)
	Opioid peptides	Exerts neuroprotective effects	β-endorphin ↑, methionine-enkephalin ↑	(48, 74)
	5-HT	Exerts neuroprotective effects	5-HT ↑, SERT ↑, 5-HT_2_A ↓	(75)
Synaptic plasticity	Synaptic reconfiguration	Restores synaptic receptor balance; upregulates proteins related to synaptic plasticity	BDNF/TrkB signaling pathway ↑	(76–81)
		Increases excitatory synapses	NGL-3/L1cam signaling pathway ↑	(82)
	Dendritogenesis	Enhances dendritic plasticity	Rho GTPase (RhoA ↓, Rac1 ↑, Cdc42 ↑)	(83–85)
			miR-134/LIMK1 axis	(86)
	Axonal regeneration	Promotes myelin repair	MBP ↑	(87)
		Facilitates growth cone formation and axonal extension	Nogo-A/NgR signaling pathway ↓, RhoA/ROCK2 signaling pathway ↓	(87–89)
			Ternary inhibitory complex (NgR ↓, p75^NTR^ ↓, LINGO-1 ↓)	(90)
			miR-132/Sox2 axis	(91)
		Promotes axonal rewiring	miR-181b/PirB axis	(92)
			PTEN/Akt/mTOR signaling pathway	(93)
Neurogenesis facilitation	Neural stem cell proliferation	Promotes neural stem cell proliferation	Notch1/Hes1 signaling pathway ↑	(94–96)
			Notch1/miR-223/PTEN axis	(97)
			ERK1/2 signaling pathway ↑	(98)
		Promotes the G1/S phase transition	p21 ↓, p27 ↓, cyclin/CDK complex ↑, Rb ↑	(95, 96, 98)
	Neural stem cell differentiation	Promotes neural stem cell proliferation and differentiation	LPA ↓, PRG5 ↑, Nogo-A ↓, RhoA ↓	(99)
			BDNF/PI3K signaling pathway ↑, VEGF/PI3K signaling pathway ↑	(100)
	Neural progenitor cell proliferation	Promotes neural progenitor cell proliferation	Wnt1/β-catenin signaling pathway ↑	(101)
	Neural stem cell differentiation into neurons	Promotes neural stem cell differentiation into neurons	miR-146b/NeuroD1 axis ↑	(102)
	Neuronal maturation	Promotes neuronal growth, migration, and maturation	NGF ↑, Gas7 ↑	(103, 104)

↑, upregulated by acupuncture; ↓, downregulated by acupuncture. 2-AG, 2-arachidonoylglycerol; 5-HT, 5-hydroxytryptamine; 5-HT_2_A, 5-HT receptor 2A; A_1_R, adenosine A_1_ receptor; AEA, anandamide; Akt, protein kinase B; BDNF, brain-derived neurotrophic factor; cAMP, cyclic adenosine monophosphate; CB_1_R, cannabinoid receptor type 1; Cdc42, cell division control protein 42; CREB, cAMP response element-binding protein; CDK, cyclin-dependent kinase; DRD2, dopamine receptor D2; ERK1/2, extracellular signal-regulated kinase 1/2; GABA, gamma-aminobutyric acid; GABA_A_Rγ2, GABA type A receptor subunit γ2; GABA_B_R, GABA type B receptor; GABAT, GABA transaminase; GAP-43, growth-associated protein 43; Gas7, growth arrest-specific 7; GLT-1, Glu transporter 1; Glu, glutamate; GluN2A, N-methyl-D-aspartate receptor subunit 2A; GluN2B, N-methyl-D-aspartate receptor subunit 2B; Grm1a, metabotropic glutamate receptor 1a; GSK-3β, glycogen synthase kinase-3β; Hes1, hairy and enhancer of split 1; KCC2, K^+^-Cl^-^ cotransporter 2; L1cam, L1 cell adhesion molecule; LIMK1, LIM domain kinase 1; LINGO-1, leucine-rich repeat and immunoglobulin domain-containing protein 1; LPA, lysophosphatidic acid; m-calpain, muscle calpain; MBP, myelin basic protein; miR, microRNA (e.g., miR-9, miR-132, miR-134, miR-146b, miR-181b, miR-223); mTOR, mechanistic target of rapamycin; NeuroD1, neurogenic differentiation factor 1; NGF, nerve growth factor; NgR, Nogo receptor; NGL-3, netrin-G ligand 3; Nogo-A, neurite outgrowth inhibitor A; Notch1, neurogenic locus notch homolog protein 1; p21, cyclin-dependent kinase inhibitor 1A; p27, cyclin-dependent kinase inhibitor 1B; p38, p38 mitogen-activated protein kinase; p75^NTR^, p75 neurotrophin receptor; PGC-1α, peroxisome proliferator-activated receptor gamma coactivator 1-α; PI3K, phosphatidylinositol 3-kinase; PirB, paired immunoglobulin-like receptor B; PKA, protein kinase A; PKCϵ, protein kinase C epsilon; PRG5, plasticity-related gene 5; PTEN, phosphatase and tensin homolog; Rac1, Ras-related C3 botulinum toxin substrate 1; Rb, retinoblastoma protein; RhoA, Ras homolog family member A; ROCK2, Rho-associated coiled-coil containing protein kinase 2; SERT, serotonin transporter; Sox2, SRY-box transcription factor 2; STAT3, signal transducer and activator of transcription 3; TrkB, tropomyosin receptor kinase B; VEGF, vascular endothelial growth factor; Wnt1, Wnt family member 1.

### 2.2 Synaptic plasticity modulation

#### 2.2.1 Synaptic reconfiguration

The dynamic changes in synaptic structure and strength, known as synaptic plasticity, represent the structural basis for the reorganization of brain function (106). After the occurrence of IS, synaptic connectivity declines in the acute phase, followed by compensatory enhancement driven by endogenous repair mechanisms during recovery (107). Electron microscopy analysis has revealed that EA improves synaptic ultrastructure by increasing the number of synapse and synaptic vesicles, as well as enhancing postsynaptic density, synaptic cleft width and synaptic interface curvature (39, 76). At the molecular level, EA activates the tissue-type plasminogen activator -mediated brain-derived neurotrophic factor/tropomyosin receptor kinase B (BDNF/TrkB) signaling pathway (77–80). This process normalizes the BDNF/pro-BDNF ratio, restores the balance of synaptic receptors (e.g., α-amino-3-hydroxy-5-methyl-4-isoxazolepropionic acid receptor, GluN1, and GABA_A_R), and upregulates plasticity-related proteins, including Ca^2+^/calmodulin-dependent protein kinase II, postsynaptic density protein 95, and Synapsin-1 (81). Additionally, EA also stimulates the netrin-G ligand 3/L1 cell adhesion molecule pathway, which increases excitatory synapse density via vesicular glutamate transporter 1 aggregation. Ultimately, this signaling axis ameliorates depression-like behaviors and motor deficits (82).

#### 2.2.2 Dendritogenesis

IS initially disrupts neural circuits by reducing dendritic spine density and arborization complexity (108). EA reverses these deficits by increasing branch density and restoring dendritic architecture (76). This structural repair is mediated by regulation of the Rho GTPase family, including Ras homolog family member A (RhoA), Ras-related C3 botulinum toxin substrate 1, and cell division control cycle 42 (83, 84). EA regulates the expression of Rho GTPase family proteins, thereby promoting spine formation and filamentous actin assembly (85). Furthermore, EA downregulates microRNA (miR)-134 and increases LIM domain kinase 1 phosphorylation. This process enhances the repair cascade and promotes dendritic spine maturation (86).

#### 2.2.3 Axonal regeneration

Following IS, axonal degeneration occurs primarily through Wallerian degeneration, wherein damaged axons and myelin sheaths undergo fragmentation (109, 110). Concurrently, astrocytes and oligodendrocytes establish “physical-chemical” inhibitory microenvironment by forming a glial scar and releasing inhibitory factors such as neurite outgrowth inhibitor A (Nogo-A) and oligodendrocyte-myelin glycoprotein (111, 112). Acupuncture downregulates Nogo-A and oligodendrocyte-myelin glycoprotein in oligodendrocytes (87–89), thereby preventing their binding to the Nogo receptor and disrupting the assembly of the ternary inhibitory complex on growth cones (90). This complex consists of Nogo receptor, p75 neurotrophin receptor, and leucine-rich repeat and immunoglobulin domain-containing protein 1. Consequently, the downstream RhoA/Rho-associated coiled-coil containing protein kinase 2 pathway is inhibited, while the expression of BDNF and growth-associated protein 43 is increased, facilitating axonal elongation and growth cone formation (88, 89). In addition, EA promotes axonal regrowth by downregulating SRY-box transcription factor 2 through miR-132 (91), and supports myelin repair by upregulating myelin basic protein in oligodendrocytes (87).

Notably, EA facilitates not only axonal sprouting but also rewiring with in the corticospinal tract. It upregulates miR-181b to suppress paired immunoglobulin-like receptor B expression and subsequent RhoA/Rho-associated coiled-coil containing protein kinase 2 signaling (92). EA also promotes axonal regeneration in the C1-C4 segments of the contralateral corticospinal tract by inhibiting phosphatase and tensin homolog (PTEN) and activating the protein kinase B (Akt)/mechanistic target of rapamycin pathway (93).

In summary, EA enhances neuroplasticity after IS by regulating the BDNF/TrkB, Rho GTPase, and multiple axon guidance signaling pathways, thereby modulating synaptic structure and function, promoting dendritic spine maturation, and facilitating axonal regeneration (**Table 1**; **Figure 1**).

### 2.3 Neurogenesis facilitation

Neural stem cells (NSCs) are primitive cells in the CNS that possess the capacity for self-renewal and multi-directional differentiation. Under ischemic conditions, NSCs change from a resting to activated state by sensing signals such as inflammatory factors, neurotrophic factors and chemokines (113, 114). This activation process relies on neurogenic locus notch homolog protein 1 (Notch1) signaling pathways to initiate proliferation, after which cells differentiate into lineage-restricted neural progenitor cells (115–118). These neural progenitor cells subsequently migrate along specific routes toward the lesion site, where they differentiate into neurons and glial cells to reconstruct damaged neural circuits (118, 119). However, owing to insufficient proliferative capacity and disrupted microenvironmental homeostasis, endogenous regeneration often fails to achieve effective neural repair (120, 121).

EA stimulates NSC proliferation in brain regions such as the subventricular zone, dentate gyrus, hippocampal CA1 area and striatum (STR) after IS, and guides NSC differentiation into functional neurons and astrocytes (94, 95, 122, 123). Furthermore, EA facilitates the migration of subventricular zone- derived NSCs along the rostral migratory stream toward the STR (122). Specifically, EA promotes the G1/S phase transition and NSC proliferation by activating the Notch1/hairy and enhancer of split 1 pathway, suppressing cyclin-dependent kinase inhibitor 1A and 1B, enhancing cyclin/cyclin-dependent kinase complex activity, and inducing retinoblastoma protein phosphorylation (94–96). Meanwhile, EA upregulates miR-223 expression and inhibits PTEN through the Notch1 pathway, thereby increasing NSC numbers (97). In addition to Notch signaling, EA also modulates neurogenesis by engaging the RhoA signaling network. RhoA binds to lysophosphatidic acid, plasticity-related gene 5, and Nogo-A to form a regulatory network. This network is critical for regulating NSC proliferation and differentiation (99). EA enhances BDNF and vascular endothelial growth factor (VEGF) signaling and triggers the phosphatidylinositol 3-kinase (PI3K) pathway, thereby driving NSC proliferation and differentiation (100). EA further activates the ERK1/2 signaling cascade, elevates cell cycle protein levels, and enhances proliferating cell nuclear antigen expression, collectively driving NSC proliferation (98).

**Figure 2:**
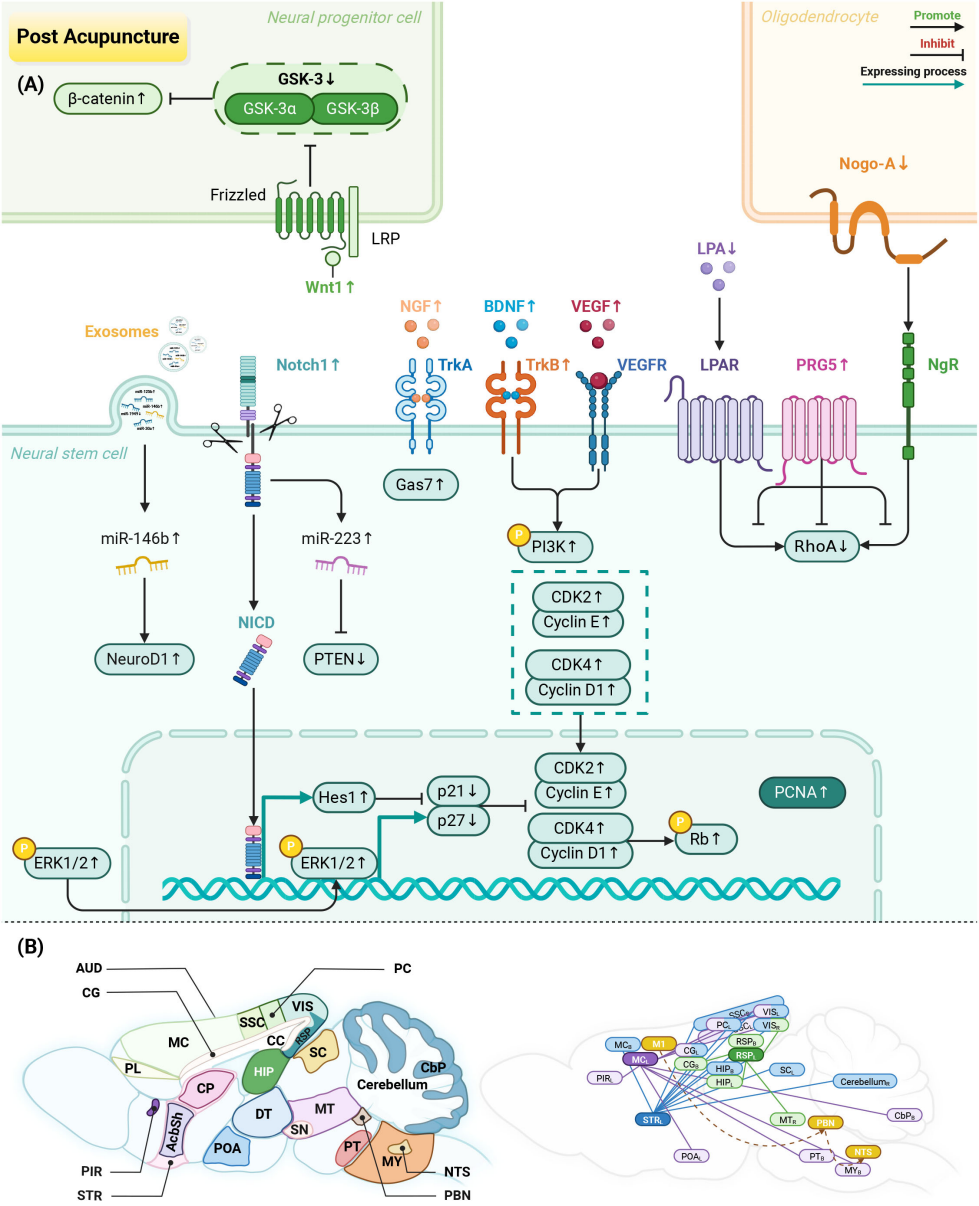
Neurogenesis and functional brain connectivity enhancement effects of acupuncture. **(A)** Neurogenesis facilitation: acupuncture activates the Notch1/Hes1 and ERK1/2 signaling pathways, reduces p21 and p27, and enhances the cyclin/CDK complex, PCNA expression, and Rb phosphorylation. These effects together advance the G1/S phase transition of the cell cycle. Acupuncture also elevates miR-223 levels and suppresses PTEN via the Notch1 pathway, thereby increasing the number of NSCs. It induces the expression of BDNF and VEGF and activates PI3K signaling, promoting the proliferation and differentiation of NSCs. Furthermore, acupuncture upregulates PRG5 and downregulates Nogo-A and LPA, synergistically inhibiting RhoA to promote NSC expansion. It stimulates the Wnt/β-catenin signaling pathway, enhancing neural progenitor cell proliferation. It increases NGF, Gas7, and the miR-146b/NeuroD1 axis to drive NSCs to differentiate into neurons. **(B)** Functional brain connectivity enhancement: key brain regions and functional connections activated by acupuncture at *Quchi* (LI11)-*Zusanli* (ST36), *Baihui* (GV20)-*Shenting* (GV24), and *Lianquan* (CV23). L: left; R: right; B: bilateral. Created in BioRender. Zhou, X. (2026) https://BioRender.com/2hytgdf.

Wnt family member 1/β-catenin signaling is the key pathway that promotes the transition of NSCs into neural progenitor cells (124). EA activates this pathway to enhance neural progenitor cell proliferation around the ischemic area (101). Regarding neuronal differentiation, EA also upregulates miR-146b levels, activates n eurogenic differentiation factor 1, and promotes NSC differentiation into neurons (102). Additionally, EA increases nerve growth factor and growth arrest-specific 7, which support neuronal growth, migration, and maturation (103, 104) (**Table 1**; **Figure 2A**).

Interestingly, EA-mediated regulation of neurogenesis follows a time-dependent pattern. During the subacute stage of IS (3–14 days), EA mainly promotes NSC proliferation and glial differentiation. In the subsequent stage (21 days), its effects shift to promoting neuronal differentiation, thereby facilitating neural circuit reorganization (95, 122). Thus, EA dynamically regulates NSC proliferation, migration, and differentiation, offering a potential approach for endogenous neural regeneration.

### 2.4 Functional brain connectivity enhancement

Beyond causing ischemic local tissue injury, IS can also disrupt the entire brain’s functional network (125). Growing evidence indicates that acupuncture can regulate functional connectivity between brain regions by engaging plasticity mechanisms, thereby promoting functional remodeling of neurons and nuclei (126, 127). Notably, research in this field has primarily focused on specific acupoint pairs. In all included studies, the ischemic lesion was induced in the left hemisphere. The acupoint pairs investigated include *Quchi* (LI11)-*Zusanli* (ST36) for motor function recovery, *Baihui* (GV20)-*Shenting* (GV24) for cognitive function improvement, and *Lianquan* (CV23) for dysphagia (**Figure 2B**).

EA at *Quchi* (LI11)-*Zusanli* (ST36) not only enhances activity in motor-related areas, including the left motor cortex (MC) and STR, as well as bilateral dorsal thalamus and somatosensory cortex (SSC), but also engages memory- and sensory-processing regions, namely the bilateral hippocampus (HIP), left corpus callosum and right auditory cortex and piriform cortex (128, 129). This multimodal engagement suggests that EA facilitates motor recovery through integrated network modulation, not merely via local effects. In parallel, these effects are further reflected in functional connectivity: EA enhances local synergistic connectivity between the left MC and some brain regions such as SSC, STR (130), while establishing long-range connections between the left STR and a distributed network encompassing sensorimotor (SSC, cerebellum) and cognitive-integration regions such as the retrosplenial cortex, cingulate gyrus, visual cortex, and HIP (131). From a global perspective, EA increases activity and connectivity within key nodes of the functional network. These include the right corpus callosum in the sensorimotor network, the left visual cortex in the sensory network, and the right MC in the salient network (132). Network analysis showed that EA restores network balance by modulating node characteristics (133). Specifically, EA strengthens information hub function by increasing the centrality of the right ventral HIP, weakens abnormal motor regulation by reducing the centrality of the right substantia nigra, and normalizes the overactive reward circuit by regulating the degree of centrality of the left nucleus accumbens shell. Furthermore, 18F-FDG/PET imaging confirmed that EA enhances glucose metabolism in caudate putamen, MC and SSC, which increases neural activity in these regions (134).

In terms of improving cognitive function after IS, EA at *Baihui* (GV20)-*Shenting* (GV24) enhanced connectivity of the core episodic memory circuit (retrosplenial cortex-HIP-cingulate gyrus), while promoting neural activity in the auditory cortex, dorsal thalamus, and sensorimotor regions (135, 136). Furthermore, proton magnetic resonance spectroscopy revealed that EA increases neurochemical metabolites (particularly N-acetylaspartic acid and choline) in the HIP and prefrontal cortex, supporting neural activity and metabolic recovery (137).

For dysphagia rehabilitation, EA at *Lianquan* (CV23) activates excitatory neurons in the fifth layer of the primary motor cortex. Signals are sent from the parabrachial nucleus to the nucleus tractus solitarius, which forms the primary motor cortex-parabrachial nucleus-nucleus tractus solitarius transsynaptic pathway (138–141). This process strengthens the swallowing reflex, thereby promoting recovery of swallowing function.

In summary, acupuncture creates a favorable molecular and cellular environment for neural repair by modulating neurotransmitters and synaptic plasticity. Concurrently, it supports structural renewal by facilitating neurogenesis. Acupuncture also improves the integration and efficiency of whole-brain networks, which is shown by enhancing functional connectivity. These mechanisms operate at the molecular, cellular, and systems levels, collectively supporting the efficacy of acupuncture in promoting neural function recovery after IS.

## 3 Mechanisms of acupuncture on endocrine modulation

Beyond its direct effects on the nervous system mentioned above, acupuncture also engages systemic hormonal pathways. The neuroendocrine system serves as a critical bridge, translating neural signals into sustained humoral responses that influence peripheral organs and overall homeostasis. This section examines how acupuncture modulates key endocrine axes and hormone signaling following IS.

### 3.1 Hypothalamic-pituitary-adrenal axis

The HPA axis is a key part of the neuroendocrine system, which contributes to keeping the body homeostasis. It regulates adaptive physiological responses via a hormonal cascade involving the amygdala, paraventricular nucleus, anterior pituitary, and adrenal cortex (142). Within the HPA axis, adrenocorticotropic hormone released from the anterior pituitary stimulates the adrenal cortex to produce cortisol, triggering a systemic stress response (143). However, under pathological stressors (such as ischemia and hypoxia), hyperfunction of the HPA axis induces metabolic disorders, immune imbalances and CNS dysfunction (144). Acupuncture can modulate HPA axis activity in multiple disease contexts (145). Acupuncture reduces serum adrenocorticotropic hormone and cortisol levels in both ischemia-reperfusion rodent models (146) and human clinical studies (147). This indicates that it may alleviate ischemia-related pathological damage by regulating the HPA axis.

### 3.2 Renin-angiotensin system

The renin-angiotensin system is the major humoral regulator of blood pressure and electrolyte homeostasis (148). Its pathophysiological effects are mediated by angiotensin II (Ang II) acting through its receptor type 1 (AT_1_R) and type 2 (AT_2_R) signaling pathways (149). Abnormal activation of the Ang II/AT_1_R axis can promote cerebral vasoconstriction and disrupt the structural and functional integrity of the blood-brain barrier. It can also trigger the release of pro-inflammatory factors and activate oxidative stress-related pathways, accelerating perivascular fibrosis (150). EA regulates the expression of Ang II in brain tissue, inhibits AT_1_R and its downstream effectors such as Gq protein, inositol trisphosphate, and diacylglycerol, and thereby reduces vasoconstriction (151). Meanwhile, EA supports AT_2_R-driven vasodilation and neuroprotection. Together, these mechanisms form a dual regulatory network that improves microcirculation in the cerebral ischemic area.

### 3.3 Insulin-like growth factor-1

Insulin-like growth factor-1 (IGF-1), a polypeptide synthesized in the liver and released into the circulation, crosses the blood-brain barrier to enter the cerebrospinal fluid and brain parenchyma. Through autocrine or paracrine mechanisms, locally produced IGF-1 is essential for maintaining cerebral homeostasis (152). In clinical settings, elevated serum IGF-1 levels during the acute phase of IS correlate with enhanced short-term neurological recovery and improved three-month survival rates; however, its prognostic value for long-term outcomes (e.g., beyond two years) remains unconfirmed (153, 154). IGF-1 confers neuroprotection after IS by modulating neurovascular function, attenuating neuroinflammation, and counteracting excitotoxicity (155). EA upregulates IGF-1 gene and protein expression in a monkey model of focal cerebral ischemia, thereby promoting functional recovery (156).

### 3.4 Erythropoietin

Erythropoietin (EPO) is a glycoprotein hormone that regulates hematopoietic function. Although EPO is synthesized in the fetal liver and adult kidneys, the brain is also an important site of EPO production. It is generated locally by several CNS cell types, including astrocytes, neurons, and endothelial cells (157, 158). EPO binds to its receptors to inhibit apoptosis, promote angiogenesis, and regulate immune responses (159). EA enhances the expression of hypoxia-inducible factor 1-alpha, activates EPO-mediated Janus kinase 2 (JAK2)/STAT3 and STAT5 cascades, thereby inhibiting neuronal apoptosis (160, 161). In addition, EA also activates EPO-dependent Src tyrosine kinase and VEGF pathways to promote angiogenesis, which establishes a favorable condition for nerve repair (162).

### 3.5 Estrogen

Sex is a key factor influencing the incidence of IS. Epidemiological data show thatpremenopausal women have a significantly lower incidence of IS than age-matched men. However, it rises sharply in postmenopausal women, who also experience poorer outcomes (163). These observations indicate that the decrease of estrogen is directly related to the increase of IS risk, supporting the potential neuroprotective effect of estrogen. Estrogen is primarily produced in the ovaries and adrenal glands. Due to its lipophilic nature, it readily crosses the blood-brain barrier and binds to estrogen receptors (ER) in the brain, which mediates the regulation of neural function (164). In an ovariectomized rat ischemia-reperfusion injury model, ER signaling is abnormal. EA restores ERβ and phosphorylated ERα (Ser118) expression, as well as regulates B-cell lymphoma 2 and cleaved cysteinyl aspartate specific proteinase-3 expression, thereby alleviating neuronal injury (165).

### 3.6 Irisin

Irisin is a cleavage product of fibronectin type III domain-containing protein 5 (166). Skeletal muscle is the main source of irisin production, contributing approximately 70% of circulating irisin (167, 168). Irisin confers neuroprotection through multiple mechanisms, including reducing oxidative stress, suppressing neuroinflammation, modulatin g blood-brain barrier permeability, and promoting stem cell differentiation (169). EA enhances fibronectin type III domain-containing protein 5 expression in both skeletal muscle and the brain, which leads to increased irisin levels. This process activates BDNF-mediated VEGF/Akt/endothelial nitric oxide synthase signaling, thereby promoting angiogenesis in ischemic areas and accelerating nerve repair (170, 171).

### 3.7 Melatonin and circadian rhythm

Melatonin is an endogenous hormone mainly synthesized by the pineal gland (172). It exerts three antioxidant effects by binding to specific receptors, including scavenging reactive oxygen species (ROS), activating endogenous oxidative systems, and enhancing mitochondrial function (173). In addition, melatonin can inhibit the activation of microglia and reduce the release of pro-inflammatory cytokines, which demonstrates a potential neuroprotective ability (174). EA enhances the expression of arylalkylamine N-acetyltransferase, thereby activating melatonin receptor 2 and the PTEN-induced putative kinase 1/parkin RBR E3 ubiquitin protein ligase signaling pathway. This process reduces ROS accumulation, inhibits NOD-like receptor family pyrin domain containing 3 (NLRP3) inflammasome activation, and improves cognitive function of IS rats (175). EA can also modulate exogenous melatonin, exerting anti-inflammatory and anti- apoptotic effects (176).

Circadian rhythm disorder is also linked to the pathological processes of IS, which is primarily driven by dysregulation of brain and muscle ARNT-like 1 and circadian locomotor output cycles kaput (177). This chronobiological dysfunction is directly linked to a key clinical observation: IS that occurs at night is often associated with a worse prognosis than IS with daytime onset (178). EA upregulates the brain and muscle ARNT-like 1/circadian locomotor output cycles kaput ratio in the cerebral tissue of MCAO model rats, reducing damage to both neurons and endothelial cells (179). Nonetheless, as rodents are nocturnal creatures, their circadian rhythms and endocrine physiology differ from those of humans. Therefore, the above experimental findings and their clinical implications should be interpreted with caution.

**Figure 3:**
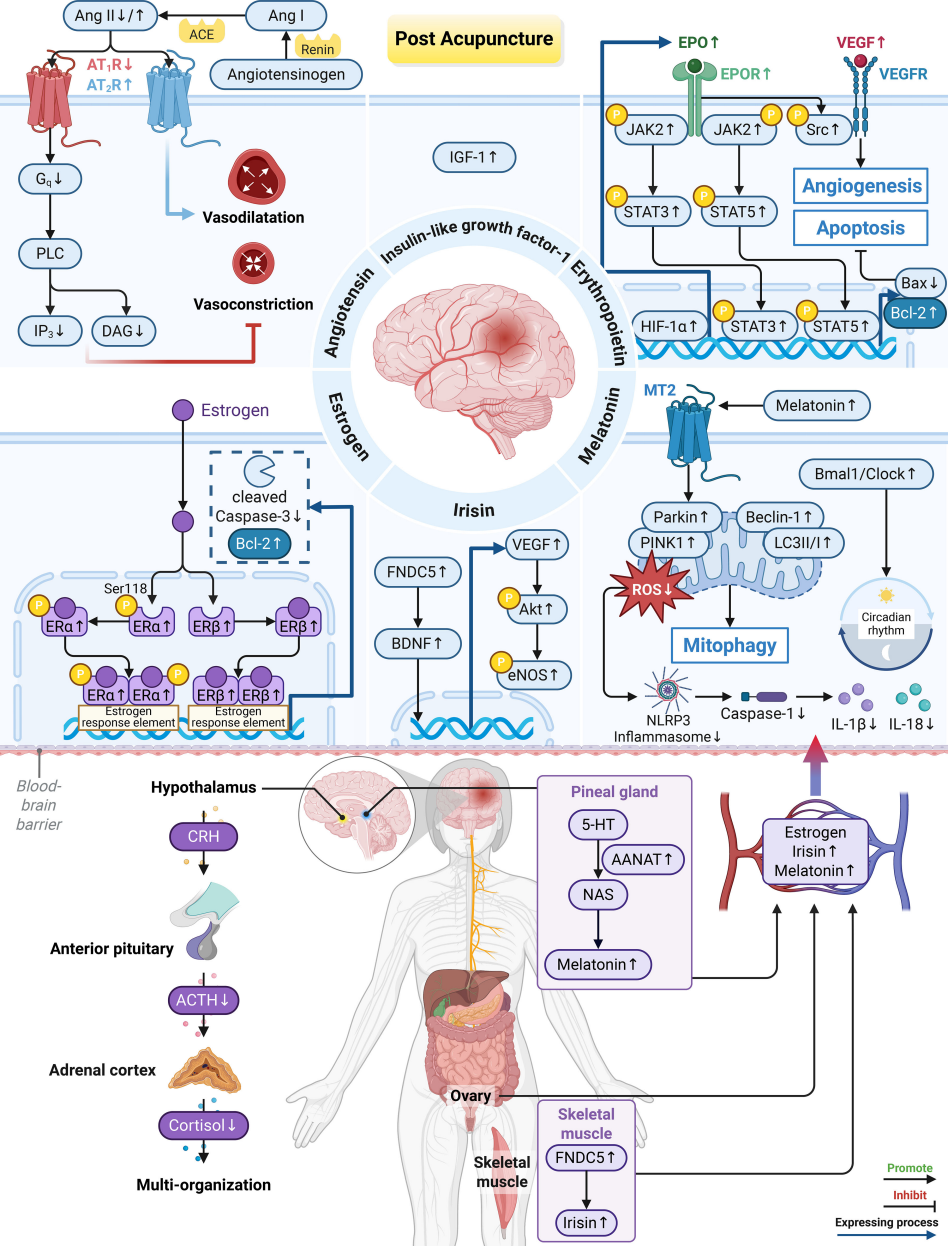
Acupuncture regulates the endocrine system to exert neuroprotective effects. (1) HPA axis: acupuncture reduces the levels of serum ACTH and cortisol, thereby attenuating HPA axis excessive activation. (2) Renin-angiotensin system: acupuncture modulates vascular function by Ang II. It suppresses AT_1_R and downstream factors (G_q_, IP_3_, DAG) while stimulating AT_2_R. (3) Erythropoietin: acupuncture enhances the HIF-1α/EPO axis to promote JAK2/STAT3 and STAT5 pathways, which inhibits apoptosis. EPO further triggers the Src signaling, which facilitates angiogenesis together with VEGF pathway. (4) Estrogen: acupuncture increases the levels of p-ERα and ERβ to exert anti-apoptotic effects. (5) Irisin: acupuncture upregulates FNDC5/Irisin expression in the skeletal muscle and brain. This activation further upregulates BDNF and the VEGF/Akt/eNOS pathways, which promotes nerve and vascular repair. (6) Melatonin: acupuncture enhances the secretion of melatonin by elevating AANAT expression in the pineal gland. Melatonin binds to MT2 receptors, activating the PINK1/Parkin pathway and promoting mitophagy. This process alleviates ROS accumulation and neuroinflammation. Acupuncture also increases the ratio of Bmal1/Clock, thereby improving circadian rhythm disorders. Created in BioRender. Zhou, X. (2026) https://BioRender.com/v2km1sg.

In summary, acupuncture confers endocrine-mediated neuroprotection by modulating multiple pathways, including the HPA axis, renin-angiotensin system, IGF-1, EPO, estrogen, irisin, and melatonin. These pathways act in concert to reduce stress hormones, balance vasomotor tone, suppress apoptosis and neuroinflammation, and promote angiogenesis and neural repair (**Table 2**; **Figure 3**).

**Table 2:** Mechanisms of acupuncture in regulating the endocrine system in ischemic stroke.

Core molecules/processes	Primary functions	Key mechanisms	Refs
HPA axis	Inhibits excessive stress response	ACTH ↓, cortisol ↓	(146, 147)
Renin-angiotensin system	Inhibits vasoconstriction; promotes vasodilation; exerts neuroprotective effects	Ang II ↓/↑, AT_1_R ↓, AT_2_R ↑, G_q_ ↓, IP_3_ ↓, DAG ↓	(151)
IGF-1	Exerts neuroprotective effects	IGF-1 ↑	(156)
EPO	Exerts anti-apoptotic effects	HIF-1α/EPO axis ↑, EPOR/JAK2/STAT3/5 signaling pathway ↑	(160, 161)
	Promotes angiogenesis	EPO-mediated Src and VEGF signaling pathways ↑	(162)
Estrogen	Exerts anti-apoptotic effects	p-ERα ↑, ERβ ↑	(165)
Irisin	Promotes neural and vascular repair	FNDC5 ↑, Irisin ↑, BDNF ↑, VEGF/Akt/eNOS signaling pathway ↑	(170, 171)
Melatonin and circadian rhythm	Promotes mitophagy; reduces neuroinflammation	AANAT ↑, Melatonin ↑, PINK1/Parkin ↑	(175)
	Ameliorates circadian rhythm disruption	Bmal1/Clock ↑	(179)

↑, upregulated by acupuncture; ↓, downregulated by acupuncture. AANAT, arylalkylamine N-acetyltransferase; ACTH, adrenocorticotropic hormone; Akt, protein kinase B; Ang II, angiotensin II; AT_1_R, Ang II type 1 receptor; AT_2_R, Ang II type 2 receptor; BDNF, brain-derived neurotrophic factor; Bmal1, brain and muscle ARNT-like 1; Clock, circadian locomotor output cycles kaput; DAG, diacylglycerol; eNOS, endothelial nitric oxide synthase; EPO, erythropoietin; EPOR, EPO receptor; ER, estrogen receptor; FNDC5, fibronectin type III domain-containing protein 5; G_q_, Gq protein; HIF-1α, hypoxia-inducible factor 1-alpha; HPA axis, hypothalamic-pituitary-adrenal axis; IGF-1, insulin-like growth factor-1; IP_3_, inositol trisphosphate; JAK2, Janus kinase 2; PINK1, PTEN-induced putative kinase 1; Parkin, parkin RBR E3 ubiquitin protein ligase; Src, Src tyrosine kinase; STAT3, signal transducer and activator of transcription 3; STAT5, signal transducer and activator of transcription 5; VEGF, vascular endothelial growth factor.

## 4 Mechanisms of acupuncture on immunoregulation

Having discussed how acupuncture restores systemic balance by regulating hormonal axes, we now turn to its impact on inflammatory mediators. The following sections detail the regulatory effects of acupuncture on central and peripheral immune cells.

### 4.1 CNS immune cells

#### 4.1.1 Microglia

Microglia, the resident immune cells of the CNS, are rapidly activated after IS and undergo significant morphological and functional changes (180). Morphologically, they shift from a ramified to an amoeboid shape. They mainly differentiate into pro-inflammatory microglia (M1 microglia) or anti-inflammatory microglia (M2 microglia) (181). Functionally, M1 microglia exacerbate neuroinflammation by releasing mediators such as tumor necrosis factor-alpha (TNF-α) and interleukin (IL)-1β. M2 microglia promote tissue repair via factors including transforming growth factor-beta, IL-10, and neurotrophic factors (182). Given the dynamic transformation of microglial phenotypes, regulating the M1/M2 balance has become a key therapeutic strategy for IS (183).

EA reduces the number of activated microglia in the ischemic penumbra and promotes their return to a resting state (184, 185). Under pathological conditions, the nuclear factor κB (NF-κB) signaling pathway plays a central role in regulating microglial polarization and neuroinflammation (186, 187). EA modulates multiple signaling cascades to inhibit NF-κB activation. It thus counteracts M1 microglia polarization and encourages M2 microglia (188–207). Additionally, EA influences SUMOylation and ubiquitination processes to interrupt NF-κB signal transduction (208, 209). Specifically, EA triggers the annexin A1/formyl peptide receptor axis, enabling SUMO-modified annexin A1 to inhibit NF-κB signaling, thereby shifting microglia toward a protective phenotype (185). It also enhances the activity of deubiquitinating enzymes in neurons and microglia, such as OTU deubiquitinase with linear linkage specificity, cylindromatosis and TNF-α-induced protein 3, along with its binding partner A20-binding inhibitor of NF-κB activation 1. These enzymes remove ubiquitin chains from NF-κB signaling components to suppress its activation (184, 210–212). Interestingly, EA engages the C-X3-C motif chemokine ligand 1/C-X3-C motif chemokine receptor 1 axis via cylindromatosis to facilitate neuron-microglia crosstalk (213). This finding seems contradictory to the aforementioned result that EA inhibits C-X3-C motif chemokine ligand 1 signal transduction (212). It is suggested that EA has a pleiotropic intervention effect in different neuroinflammatory stages or microenvironments, but its specific mechanism still needs to be further clarified.

Epigenetically, EA upregulates miR-9 to inhibit NF-κB nuclear translocation (214), and downregulates long non-coding RNA (lncRNA) 826 to modulate the Hippo pathway (215). These processes contribute to regulating microglial polarization. EA also reduces the N^6^-methyladenosine methylation of the lncRNA H19, thereby suppressing the sphingosine-1-phosphate receptor 2/Toll-like receptor 4 (TLR4)/NLRP3 signaling pathway (216). In addition, EA inhibits purinergic receptors P2X7 and P2Y1 in both microglia and astrocytes (217). And it further regulates nuclear factor erythroid 2-related factor 2 and NLRP3 inflammasome activation through purinergic receptors P2X7 (218–220).

Beyond modulating microglial polarization, EA also influences microglial survival and clearance functions. It suppresses pyroptosis by the RhoA/pyrin/gasdermin D and NLRP3 pathways (219, 221). In parallel, EA increases levels of ATP-binding cassette transporter A1 to promote efferocytosis in M2 microglia, facilitating clearance of damaged cells (222).

**Figure 4:**
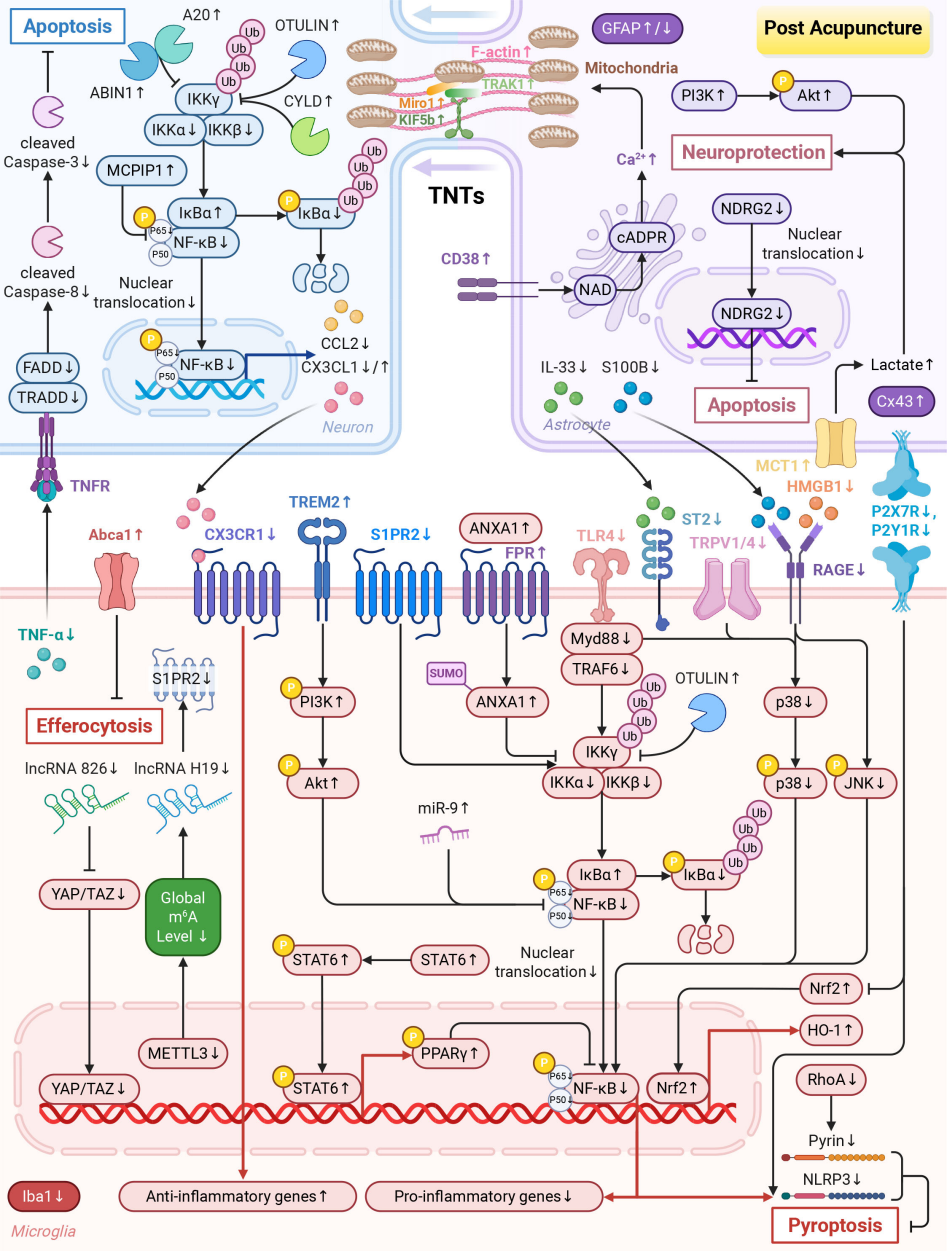
Acupuncture modulates glial cells to regulate neuroinflammation. (1) Microglia: acupuncture suppresses the TLR4/Myd88, TRPV1/4, and HMGB1/RAGE signaling pathways, activates the TREM2/PI3K/Akt, STAT6/PPARγ, ANXA1/FPR, and MCPIP1 pathways, as well as enhances the activity of deubiquitinating enzymes (OTULIN, CYLD and A20) and upregulates the related protein ABIN1. These effects together alleviate the polarization imbalance of microglia mediated by NF-κB. Additionally, acupuncture downregulates P2X7R and P2Y1R expression. This inhibition subsequently triggers the Nrf2/HO-1 pathway and inhibits NLRP3 inflammasome, thereby attenuating neuroinflammation. At the epigenetic level, acupuncture upregulates miR-9 levels to inhibit NF-κB nuclear translocation. It reduces the activity of METTL3 to lower m^6^A levels, thereby suppressing the lncRNA H19-mediated S1PR2/TLR4/NLRP3 signaling pathway. It also downregulates lncRNA 826 to modulate the Hippo pathway, thereby relieving neuroinflammation. Furthermore, acupuncture inhibits pyroptosis via the RhoA/pyrin pathway and NLRP3 inflammasome. It improves M2 microglia efferocytosis by upregulating Abca1. (2) Astrocytes: acupuncture enhances the syncytial network by promoting Cx43 expression. It can inhibit the IL-33/ST2, S100B/RAGE, and death receptor apoptotic pathways, which alleviates negative effects of astrocyte-microglia interactions. Acupuncture activates the PI3K/Akt pathway to exert neuroprotective effects. Acupuncture also inhibits apoptosis by reducing the level of NDRG2. In addition, acupuncture triggers the CD38/cADPR/Ca^2+^ signal axis to promote the transfer of mitochondria to neurons via TNTs. Finally, acupuncture improves lactate metabolism by increasing the expression of MCT1, providing energy support for neurons. Created in BioRender. Zhou, X. (2026) https://BioRender.com/zic6t6t.

In summary, EA orchestrates microglial responses through multiple mechanisms, encompassing polarization regulation centered on NF-κB, modulation of epigenetic and purinergic pathways, and survival control. Through these mechanisms, EA alleviates neuroinflammation and promotes repair after IS (**Table 3**; **Figure 4**).

**Table 3:** Mechanisms of acupuncture in regulating the immune system in ischemic stroke.

Category	Core cells	Primary functions	Key mechanisms	Refs
CNS immune cells	Microglia	Inhibits microglial activation; regulates microglial polarization; reduces neuroinflammation	TLR4/NF-κB/NLRP3 signaling pathway ↓	(188–196, 216)
			TREM2/PI3K/Akt signaling pathway ↑	(197, 198)
			HMGB1/RAGE/JNK signaling pathway ↓	(199–202)
			TRPV1/4/p38 signaling pathway ↓	(203–207)
			STAT6/PPARγ signaling pathway ↑	(206, 207)
			ANXA1/FPR axis ↑	(185)
			OTULIN ↑, A20 ↑, ABIN1 ↑	(184, 210–212)
			CYLD/CX3CL1/CX3CR1 signaling pathway	(212, 213)
			miR-9/NF-κB axis	(214)
			Hippo signaling pathway ↓	(215)
			Purinergic signaling pathway ↓	(217–220)
		Inhibits microglial pyroptosis	NLRP3/Caspase-1/GSDMD signaling pathway ↓	(219)
			RhoA/pyrin/GSDMD signaling pathway ↓	(221)
		Enhances efferocytosis in M2 microglia	Abca1 ↑	(222)
	Astrocytes	Enhances syncytial network	Cx43 ↑	(39)
		Promotes astrocytic activation	PI3K/Akt signaling pathway ↑	(223)
		Inhibits neuronal apoptosis mediated by astrocyte-neuron crosstalk	S100B/RAGE/p38/NF-κB signaling pathway ↓, TNF-α/TRADD/FADD/Caspase-8/Caspase-3 signaling pathway ↓	(224)
		Suppresses inflammatory cascade in astrocyte-microglia crosstalk	IL-33/ST2 axis ↓	(225, 226)
		Promotes lactate metabolism	MCT1 ↑	(227)
		Exerts anti-apoptotic effects	NDRG2 ↓	(228)
		Promotes functional mitochondria transfer to neurons	CD38/cADPR/Ca^2+^ signaling pathway ↑	(229)
Peripheral immune populations	Neutrophils	Reduces neutrophil infiltration into ischemic regions; inhibits neutrophil extracellular traps formation	MPO ↓, MMP-9 ↓, TIMP-1 ↑, TIMP-2 ↑	(230–234)
	Monocytes /Macrophages	Reduces monocyte infiltration into ischemic regions	CCL2 ↓	(235)
	Natural killer cells	Reduces Natural killer cell infiltration into ischemic regions; increases peripheral blood Natural killer cell counts	CCL2 ↓, NKG2D ↓, GzmB ↓, IFN-γ ↓	(196, 236)

↑, upregulated by acupuncture; ↓, downregulated by acupuncture. A20, TNF alpha-induced protein 3; ABIN1, A20-binding inhibitor of NF-κB activation 1; Abca1, ATP-binding cassette transporter A1; Akt, protein kinase B; ANXA1, annexin A1; CCL2, C-C motif chemokine ligand 2; CD38, cluster of differentiation 38; cADPR, cyclic ADP-ribose; Caspase-1, cysteinyl aspartate specific proteinase - 1; Caspase-3, cysteinyl aspartate specific proteinase -3; Caspase-8, cysteinyl aspartate specific proteinase -8; CNS, central nervous system; Cx43, connexin 43; CX3CL1, C-X3-C motif chemokine ligand 1; CX3CR1, C-X3-C motif chemokine receptor 1; CYLD, cylindromatosis; FADD, Fas-associated protein with death domain; FPR, formyl peptide receptor; GSDMD, gasdermin D; GzmB, granzyme B; HMGB1, high mobility group box 1; IFN-γ, interferon-gamma; IL-33, interleukin-33; JNK, c-Jun N-terminal kinase; MCT1, monocarboxylate transporter 1; miR-9, microRNA-9; MMP-9, matrix metalloproteinase-9; MPO, myeloperoxidase; NDRG2, n-myc downstream regulated gene 2; NF-κB, nuclear factor-kappa B; NKG2D, natural killer group 2D; NLRP3, NOD-like receptor family pyrin domain containing 3; OTULIN, OTU deubiquitinase with linear linkage specificity; p38, p38 mitogen-activated protein kinase; PI3K, phosphatidylinositol 3-kinase; PPARγ, peroxisome proliferator-activated receptor gamma; RAGE, receptor for advanced glycation end products; RhoA, Ras homolog family member A; S100B, S100 calcium-binding protein B; STAT6, signal transducer and activator of transcription 6; ST2, growth stimulation-expressed gene 2; TIMP, tissue inhibitor of metalloproteinase; TLR4, Toll-like receptor 4; TNF-α, tumor necrosis factor-alpha; TRADD, TNFRSF1A-associated via death domain; TREM2, triggering receptor expressed on myeloid cells 2; TRPV1/4, transient receptor potential vanilloid 1/4.

#### 4.1.2 Astrocytes

Astrocytes are the most abundant cell type in the CNS. They not only provide structural and metabolic support to neurons but also play a regulatory role in immune homeostasis (237). Connexin 43 participates in the formation of syncytial networks, which enable rapid communication between astrocytes (238). Similar to microglia, astrocytes can also differentiate into distinct phenotypes, thereby engaging in the immune-inflammatory processes (239).

EA exerts bidirectional regulatory effects on astrocytes (223, 224, 240–242). EA enhances the syncytial network by promoting connexin 43 expression (39), and activates the PI3K/Akt pathway to promote neural repair (223). Moreover, EA can inhibit the RAGE/p38/NF-κB and death receptor pathways by reducing levels of S100 calcium-binding protein B (224). Interestingly, EA also modulates the inflammatory cascade by inhibiting the IL-33/growth stimulation-expressed gene 2 signaling axis that mediates the inflammatory cascade response, thereby reducing the negative effects of astrocyte - microglia crosstalk (225, 226).

The Myc family plays a key role in energy metabolism. Among them, c-Myc can induce the expression of monocarboxylate transporter 1, which is responsible for transporting the energy substrates lactate and pyruvate (243). EA enhances the expression of monocarboxylate transporter 1 and promotes lactate metabolism in astrocytes, thereby providing energy substrates to neurons (227). Furthermore, n-Myc downstream regulated gene 2 is primarily expressed in astrocytes. EA reduces its expression and nuclear translocation, exerting anti-apoptotic effects (228). EA can also activate the cluster of differentiation (CD) 38/cyclic ADP-ribose/Ca^2+^ pathway in astrocytes, facilitating the transfer of functional mitochondria to neurons via tunnelling nanotubes (229). This process not only enhances neuronal energy metabolism but also prevents neuronal apoptosis, revealing a novel mechanism for EA-mediated neuroprotection (**Table 3**; **Figure 4**).

### 4.2 Peripheral immune populations

#### 4.2.1 Neutrophils

As the “mobile infantry” of the immune system, neutrophils are the first peripheral immune cells to infiltrate and accumulate in the peri-infarct region following IS (244). They mainly participate in IS pathology by releasing neutrophil extracellular traps (NETs). NETs are web-like structures composed of DNA, histones, proteases, and other components (245). Of these, myeloperoxidase is the core structural protein of NETs, while matrix metalloproteinase-9 is the key mediator promoting NET formation (246). Importantly, this process can further promote thrombosis and exacerbate neurological impairment (247). EA reduces neutrophil infiltration and downregulates the expression of myeloperoxidase and matrix metalloproteinase-9 in ischemic tissue (230–232). Additionally, EA increases levels of tissue inhibitor of metalloproteinase 1 and 2 and changes the transcription of matrix metalloproteinase-9 and tissue inhibitor of metalloproteinase 2 through histone acetylation. These mechanisms together reduce neuroinflammation and preserve blood-brain barrier integrity (233, 234).

#### 4.2.2 Monocytes/macrophages

Monocytes, the largest group of white blood cells in peripheral blood, constantly patrol the cerebral border regions under physiological conditions, providing constant immune surveillance for the CNS (248). After IS, monocytes are widely recruited to the brain under the guidance of chemokines and differentiate into macrophages and other immune cells, playing a key role in the process of immune inflammation (249). The chemokine C-C motif chemokine ligand 2 (CCL2) regulates the recruitment of monocytes/macrophages, activated T cells, and natural killer cells (NK cells), promoting their migration into the CNS (250). EA activates the expression of monocyte chemotactic protein-induced protein 1, which inhibits the NF-κB pathway and reduces CCL2 release (235). This process increases the neuroprotective effect by reducing monocyte chemotaxis and their infiltration in ischemic areas.

#### 4.2.3 Natural killer cells

NK cells are important innate immune system effector cells that mediate both immunomodulatory and direct cytotoxic killing (251). Following IS, NK cell counts and activity rise in brain tissue while falling in peripheral blood (252). Interestingly, EA regulates this pathological process in both directions. EA downregulates CCL2 expression through STAT3 signaling, which reduces NK cell infiltration into ischemic regions. EA also decreases the expression of the activating receptor natural killer group 2D on infiltrating NK cells. This process inhibits cytotoxic mediator granzyme B transcription and interferon-gamma production, thereby reducing neuronal injury (236). In addition, EA increases the proportion of CD3^-^CD49b^+^ NK cells in peripheral blood (196). This bidirectional regulation of NK cells highlights the synergistic therapeutic effects of acupuncture on the immune inflammation of IS.

**Figure 5:**
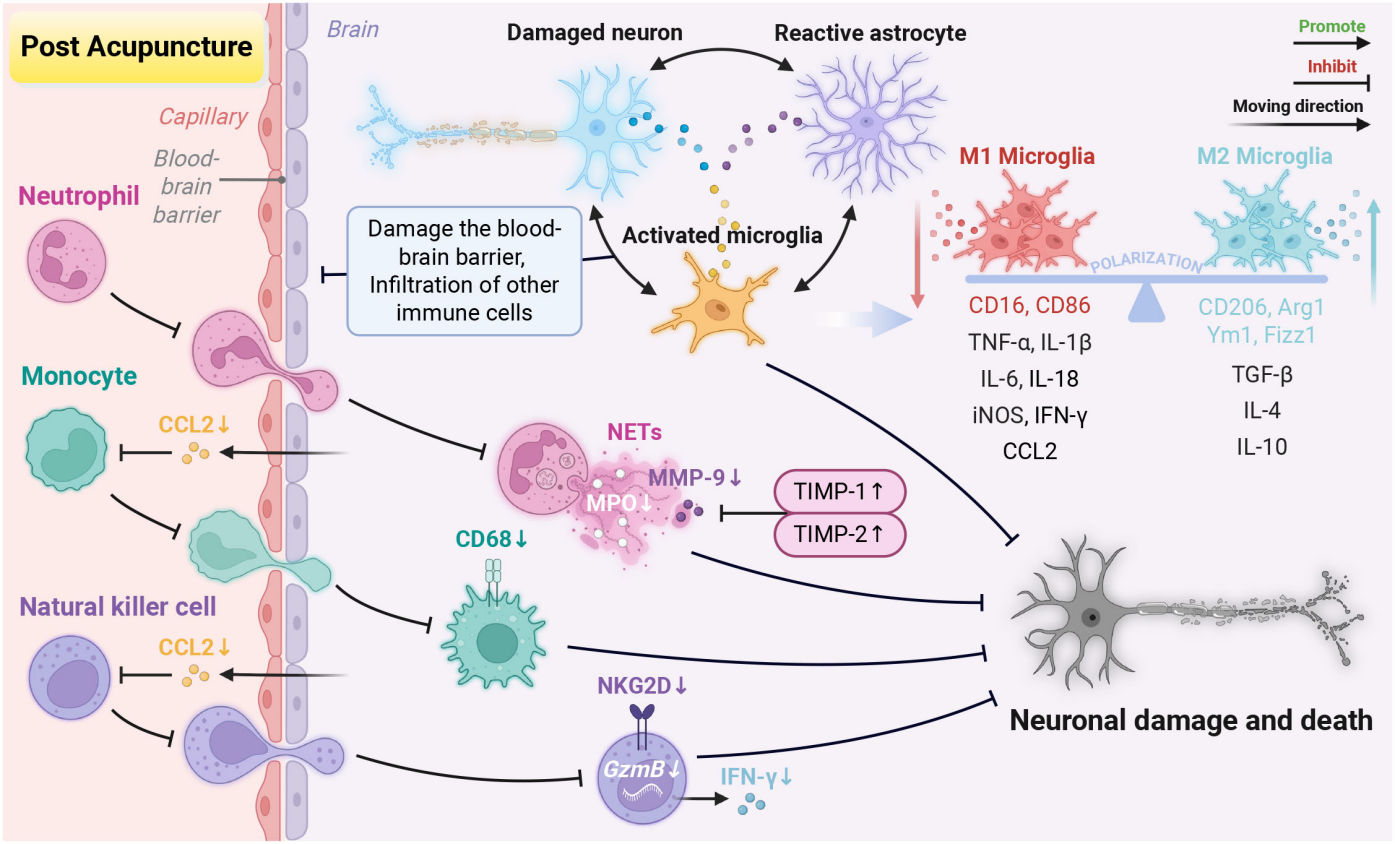
Acupuncture modulates the central and peripheral immune cells to restore immune microenvironment homeostasis. (1) CNS immune cells: acupuncture suppresses inflammatory mediators released by damaged neurons, reactive astrocytes, and activated microglia. This alleviates neuroinflammation and preserves blood-brain barrier integrity. Acupuncture modulates microglial polarization. It inhibits the M1 microglia by downregulating markers CD16, CD86, and pro-inflammatory factors (e.g., TNF-α, IL-1β, IL-6, IL-18, iNOS, IFN-γ, CCL2). Simultaneously, it promotes polarization toward the M2 microglia by upregulating markers (e.g., CD206, Arg1, Ym1, Fizz1), along with anti-inflammatory factors TGF-β, IL-4, and IL-10. (2) Peripheral immune populations: acupuncture increases TIMP-1/2 while decreasing MPO and MMP-9 expression, thereby inhibiting NETs formation. Additionally, acupuncture reduces recruitment of monocytes/macrophages and NK cells to ischemic areas by lowering CCL2. It also relieves the neurotoxic effects of NK cells by downregulating the expression of NKG2D, GzmB, and IFN-γ expression. The above mechanisms collectively counteract neuronal damage and death. Created in BioRender. Zhou, X. (2026) https://BioRender.com/opvkr9q.

In summary, in terms of the central immune system, acupuncture regulates microglial polarization, cell death and efferocytosis, controls astrocyte behavior, and reduces the negative effects of astrocyte-microglial interactions. In the peripheral immune system, acupuncture inhibits the activation of neutrophils, monocytes/macrophages, and NK cells, and prevents their infiltration into brain regions (**Table 3**; **Figure 5**). It is worth noting that during IS, thrombosis and inflammation reinforce each other, forming a key thrombo-inflammation axis (253). However, current research has overlooked how acupuncture regulates the interaction between thrombus and immune cells (19), which deserves attention in subsequent studies.

## 5 Integrated mechanisms of acupuncture on NEI network modulation

After separately exploring the regulatory effects of acupuncture on the nervous, endocrine and immune systems, it is evident that these systems do not operate in isolation. Rather, they form an integrated NEI network with intercommunication among its components. The following section synthesizes these findings to explore how acupuncture orchestrates this multi-system crosstalk—particularly through the cholinergic anti-inflammatory pathway and the brain-gut axis—to promote coordinated recovery after IS.

### 5.1 Cholinergic anti-inflammatory pathway

The cholinergic anti-inflammatory pathway is a key hub for NEI network integration, featuring multi-layered signaling and cellular responses (254). The vagus nerve transmits peripheral inflammatory signals to the CNS, activating the cholinergic anti-inflammatory pathway and releasing ACh (255). By binding to muscarinic acetylcholine receptor (mAChR) and nicotinic acetylcholine receptor (nAChR) on immune cell surfaces, ACh inhibits pro-inflammatory cytokines, forming a key neuro-immune regulation circuit (256). Clinical studies have shown that abnormal function of the cholinergic anti-inflammatory pathway is associated with IS progression, highlighting its potential as a therapeutic target (257, 258).

The cholinergic anti-inflammatory pathway is not only a neuro-immune reflex pathway but also a multi-level signal transduction network. Acupuncture increases α7nAChR levels in ischemic tissues, activates the JAK2/STAT3 pathway, and inhibits HMGB1 and NLRP3 inflammasome activation (259–261). This combined effect balances pro- and anti-inflammatory mediators, reduces abnormal microglial polarization, and ultimately alleviates neuroinflammatory damage (262, 263). EA also stimulates the brainstem parasympathetic nuclei, enhancing cholinergic signaling (including ChAT, α7nAChR, and mAChR M1-M5). This process drives three protective mechanisms: improving ischemia-reperfusion injury, inhibiting inflammatory responses, and resisting oxidative stress damage (264). Importantly, this central regulatory effect can further act on the ChAT/α7nAChR pathway in the gastrointestinal tract via the vagus nerve, upregulating serum levels of vasoactive intestinal peptide and motilin, and attenuating post-stroke inflammation (265, 266). This mechanism reflects the cross-organ protective characteristics of acupuncture on the NEI network.

In addition, acupuncture also affects cerebral vasculature and neurons by regulating the cholinergic anti-inflammatory pathway. EA increases cortical ACh release by activating cholinergic projections from the basal forebrain Meynert nucleus to the cortex. This dilation of cerebellar meningeal collateral vessels reduces vascular resistance via the mAChR M3/endothelial nitric oxide synthase pathway, which improves perfusion in the ischemic area (267, 268).

**Figure 6:**
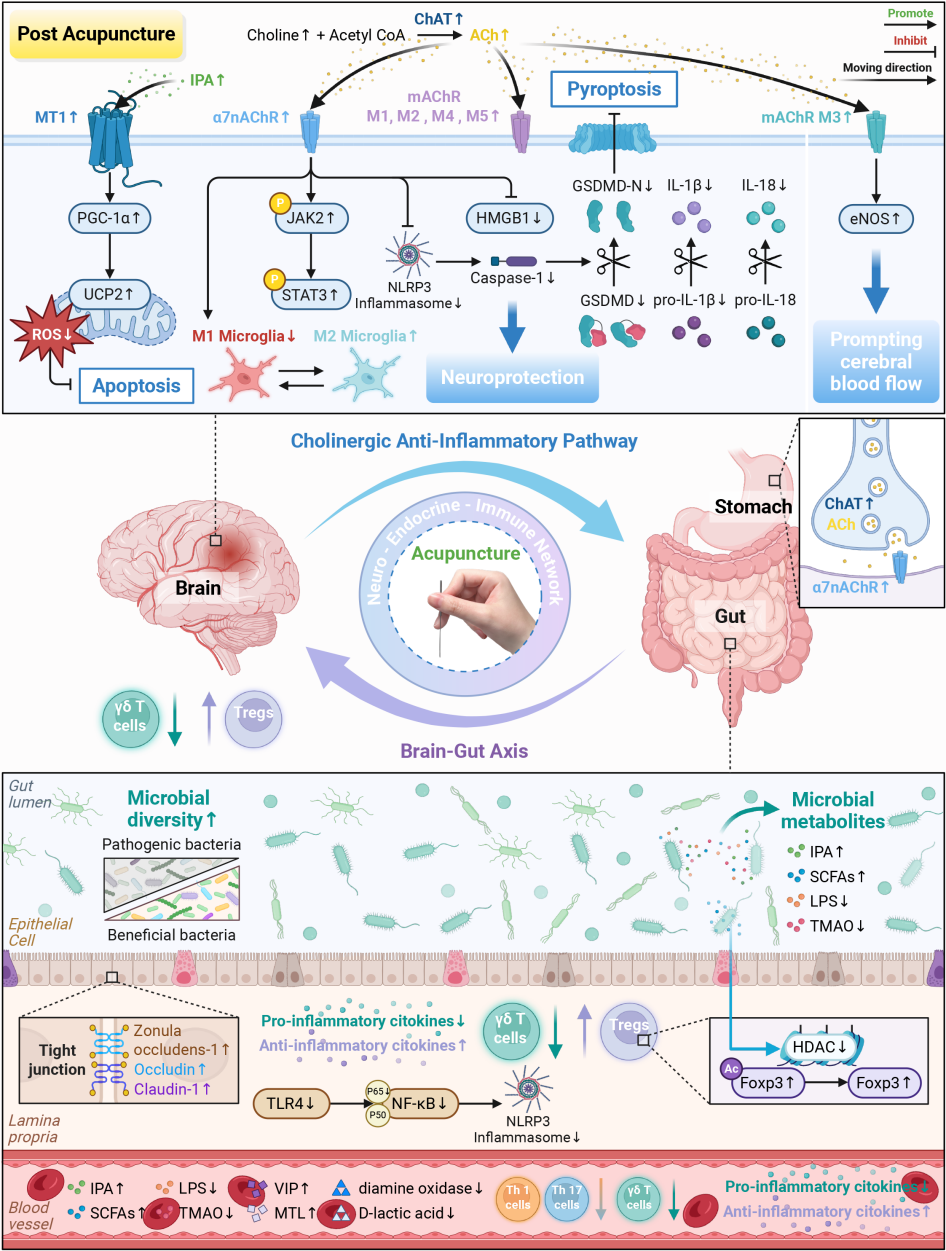
Acupuncture coordinates the cholinergic anti-inflammatory pathway and the brain-gut axis mechanism. (1) Cholinergic anti-inflammatory pathway: acupuncture upregulates the key components of this pathway in the CNS (choline, ChAT, ACh, α7nAChR, and mAChR M1-M5), exerting multiple protective effects. On the one hand, acupuncture regulates α7nAChR, activates the JAK2/STAT3 signal, inhibits NLRP3 inflammasome and HMGB1, and reduces abnormal microglial polarization. Meanwhile, this central regulatory effect can further act on the ChAT/α7nAChR pathway in the gastrointestinal tract through the vagus nerve, upregulate the levels of VIP and MTL in the serum, and improve the inflammation after IS. On the other hand, acupuncture stimulates eNOS through mAChR M3, promoting cerebral vasodilation and blood perfusion. (2) Brain-gut axis: acupuncture repairs the intestinal barrier by upregulating the expression of tight junction proteins (Zonula occludens-1, Occludin, and Claudin-1), downregulating the levels of intestinal barrier injury markers (diamine oxidase and D-lactic acid), and inhibiting the TLR4/NF-κB/NLRP3 pathway. Similarly, acupuncture promotes a shift toward beneficial microbial taxa and broadens gut microbial diversity, thereby regulating key metabolites (IPA, SCFAs, LPS, and TMAO). Among these metabolites, acupuncture increases the production of IPA, which enters the circulation and binds to MT1 in the brain, thereby activating the PGC-1α/UCP2 pathway. Acupuncture promotes the synthesis of SCFAs, inhibits HDAC activity, and increases the acetylation of Foxp3, thereby enhancing the Treg differentiation in the brain and intestine. In addition, acupuncture can inhibit the migration of gut-derived γδ T cells to the brain and weaken the pro-inflammatory activity of peripheral Th 1/17 cells, thereby establishing an anti-inflammatory cascade from the intestine to the CNS. Created in BioRender. Zhou, X. (2026) https://BioRender.com/r01oe8d.

In summary, acupuncture initiates neural protective signals via the cholinergic anti-inflammatory pathway (**Table 4**; **Figure 6**). This pathway links immune and endocrine responses through gut hormones, presenting a multi-system regulation framework. However, existing research has mainly focused on acupuncture’s effects on the central cholinergic system. Future studies should adopt an integrated circuit perspective to fully clarify the role of acupuncture in the NEI network.

**Table 4:** Mechanisms of acupuncture in regulating the neuro-endocrine-immune network in ischemic stroke.

Core molecule/process	Primary functions	Key mechanisms	Refs
Cholinergic anti-inflammatory pathway	Regulates microglial polarization; reduces neuroinflammation; reduces oxidative damage; exerts neuroprotective effects	ChAT ↑, α7nAChR/JAK2/STAT3 signaling pathway, ↑, NLRP3 ↓, HMGB1 ↓, mAChR M1-M5 ↑	(259–264)
	Modulates neuro-endocrine-immune network interactions	ChAT/α7nAChR signaling pathway, ↑, VIP ↑, MTL ↑	(265, 266)
	Promptes vasodilation and cerebral blood flow	ACh ↑, mAChR M3/eNOS signaling pathway, ↑	(267, 268)
Brain-gut axis	Reduces neuroinflammation; repairs the gut barrier	TLR4/NF-κB/NLRP3 signaling pathway, ↓, Zonula occluden-1 ↑, Occludin ↑, Claudin-1 ↑, diamine oxidase ↓, D-lactic acid ↓	(269, 270)
	Promotes beneficial shift in gut microbiota; regulates gut microbial metabolites	Microbial diversity ↑, Beneficial microbial taxa ↑, IPA ↑, SCFAs ↑, LPS ↓, TMAO ↓	(269, 271–274)
	Modulates neuro-endocrine network interactions; Exerts anti-apoptotic effects	IPA ↑, MT1 ↑, PGC-1α/UCP2 signaling pathway, ↑	(274)
	Establishes gut-to-brain anti-inflammatory cascades	SCFAs ↑, HDAC ↓, Foxp3 ↑, gut-derived Tregs ↑; inhibits migration of gut-derived γδ T cells to the brain; suppresses peripheral Th1/17 cell activity	(196, 225, 275–277)

↑, upregulated by acupuncture; ↓, downregulated by acupuncture. α7nAChR, α7 nicotinic acetylcholine receptor; ACh, acetylcholine; ChAT, choline acetyltransferase; eNOS, endothelial nitric oxide synthase; Foxp3, forkhead box P3; HDAC, histone deacetylase; HMGB1, high mobility group box 1; IPA, indole-3-propionic acid; JAK2, Janus kinase 2; LPS, lipopolysaccharide; mAChR, muscarinic acetylcholine receptor; MT1, melatonin receptor 1; MTL, motilin; NF-κB, nuclear factor-kappa B; NLRP3, NOD-like receptor family pyrin domain containing 3; PGC-1α, peroxisome proliferator-activated receptor gamma coactivator 1-alpha; SCFAs, short-chain fatty acids; STAT3, signal transducer and activator of transcription 3; Th cells, T helper cells; TLR4, Toll-like receptor 4; TMAO, trimethylamine N-oxide; Tregs, regulatory T cells; UCP2, uncoupling protein 2; VIP, vasoactive intestinal peptide; γδ T cells, gamma delta T cells.

### 5.2 Brain-gut axis

The brain-gut axis includes communication among the gut microbiota, their metabolites, and endocrine signals. This interaction extends beyond local organs to form a body-wide regulatory network. EA suppresses TLR4/NF-κB/NLRP3 pathway activation, increases levels of tight junction proteins (Zonula occluden-1, Occludin, and Claudin-1), and reduces serum markers of intestinal barrier injury (diamine oxidase and D-lactic acid), which establishes a structural basis for repairing the damaged intestinal barrier (269, 270). Notably, the gut microbiota is crucial for activating this axis. Their metabolites not only interact with enteric nerve receptors but also transmit signals to the brain via the vagus nerve and reach distant organs through the circulation, which influences the pathological process of IS (278, 279).

Functionally, acupuncture promotes a shift in gut microbiota composition toward a more beneficial profile. Acupuncture alters important gut-brain metabolites, such as indole-3-propionic acid, short-chain fatty acids, lipopolysaccharide, and trimethylamine N-oxide (269, 271–274). EA promotes the release of indole-3-propionic acid that binds to melatonin receptor 1 in the brain, activating the peroxisome proliferator-activated receptor gamma coactivator 1-alpha/uncoupling protein 2 signaling pathway (274). Furthermore, EA enhances the production of short-chain fatty acids, inhibits histone deacetylase activity, and increases acetylation of forkhead box P3, which regulates the differentiation and function of regulatory T cells in both the brain and gut (275, 276). Interestingly, EA also inhibits gut-derived gamma delta T cell migration to the brain and attenuates the pro-inflammatory activity of peripheral T helper 1/17 cells, thereby establishing an anti-inflammatory cascade from the gut to the CNS (196, 225, 277).

In summary, acupuncture forms an integrated regulatory network by modulating neural signals, endocrine hormones, and immune responses (**Table 4**; **Figure 6**). Through the brain-gut axis, acupuncture further modulates the intestinal microbiota and their metabolites, which achieves integrated immune regulation across functional axes (pro-inflammatory and anti-inflammatory balance) and spatial domains (brain, gut, and circulation). This multi-dimensional regulatory mechanism highlights the comprehensive ability of acupuncture in treating IS.

## 6 Mechanisms supported by clinical evidence

Building on the preceding overview that primarily derived from animal studies—this section now shifts to acupuncture-related mechanisms supported by clinical evidence. Specifically, we examine how acupuncture modulates systemic function at the human level through three interrelated dimensions: circulating biomarkers, neuroimaging, and intestinal microecology. Together, these perspectives offer a more holistic and multidimensional understanding of its therapeutic mechanisms.

At the nervous system level, acupuncture alleviates sympathetic hyperactivity by reducing heart rate variability in patients with insomnia after IS (280). Its analgesic effect may be related to increased release of endogenous opioid peptides (α-endorphin, β-endorphin, enkephalin, dynorphin) in plasma and decreased levels of 5-HT, dopamine and substance P (281, 282). Additionally, acupuncture upregulates serum levels of BDNF, neuron-specific enolase, S100 calcium-binding protein B, VEGF, endothelin, thromboxane B2 and 6-keto-prostaglandin F1α, promoting neurovascular unit repair (283–286). Functional magnetic resonance imaging studies further revealed the neural basis through which acupuncture regulates post-stroke complications. In terms of motor function recovery, scalp acupuncture provides potential imaging biomarkers for motor dysfunction by regulating theamplitude of low frequency fluctuations of the ipsilateral anterior central gyrus and posterior central gyrus (287). For hemiplegic patients, EA can downregulate the functional connectivity of the ipsilateral parietal network and enhance the small-world attributes of the whole brain network (288). In terms of sensory dysfunction, EA activates the contralateral primary SSC, bilateral secondary SSC and ipsilateral cerebellum of patients, suggesting that the compensatory recruitment of the sensorimotor cortex may be the neural basis for acupuncture to promote sensory recovery (289). In terms of post-stroke sleep disorders, EA improves patients’ sleep and cognitive functions by inhibiting the activity of key nodes in the default mode network, enhancing the connection of cognitive control networks, and regulating the functional lateralization of the cerebral hemispheres (290). It is worth noting that reduced acupuncture reactivity after IS may be related to changes in neural plasticity after injury (291), and different acupoint selections can activate specific brain regions (292).

At the endocrine system level, acupuncture increases levels of somatostatin in patients’ cerebrospinal fluid and plasma (293). This hypothalamic hormone inhibits growth hormone release, modulates neurotransmission, and exerts anti-inflammatory effects (294, 295). Moreover, acupuncture decreases serum levels of N-terminal pro-B-type natriuretic peptide (296). The peptide, an inactive precursor fragment, aids in differentiating stroke subtypes (297, 298).

At the immune system level, acupuncture upregulates serum IL-10 and IL-12 p70 in patients (299, 300), and downregulates TNF-α, IL-1β, IL-6, IL-17 and high-sensitivity C-reactive protein (301–303). Simultaneously, it reduces levels of intestinal fatty acid-binding protein, D-lactate, lipopolysaccharide, and lipopolysaccharide binding protein, and decreases peripheral blood neutrophil counts, thereby inhibiting the systemic inflammatory response after IS (296).

At the brain-gut axis level, acupuncture increases the relative abundance of beneficial bacteria (such as *Bifidobacterium* and *Lactobacillus*), reduces that of harmful bacteria (such as *Escherichia coli* and *Enterococcus*) (296, 304, 305), downregulates trimethylamine N-oxide levels (303), and upregulates vasoactive intestinal peptide content in cerebrospinal fluid (306). It has been confirmed that acupuncture alleviates CNS inflammation by modulating gut microbiota composition, structure and metabolite levels.

In conclusion, clinical research evidence demonstrates that acupuncture promotes functional recovery after IS through the NEI network. This is supported by functional brain reorganization observed in neuroimaging, shifts in circulating hormones and cytokines, and improvements in intestinal microecological community structure. Therefore, this multi-dimensional effect constitutes the comprehensive biological basis for the therapeutic effect of acupuncture.

## 7 Conclusions and future perspectives

Acupuncture has been inscribed on the United Nations Educational, Scientific and Cultural Organization’s Intangible Cultural Heritage List, marking its medical value and cultural significance (307–310). Although this empirical medical intervention has clinical efficacy, more multidisciplinary research integration is still needed to promote the scientific nature of its theory and the standardization of its methods (311). This review systematically investigated how acupuncture affects the recovery of IS based on the NEI network. As mentioned above, acupuncture does not target isolated targets but acts on multi-level targets to exert therapeutic effects, suggesting acupuncture’s potential in treating IS and its holistic regulatory characteristic.

Parameter selection is crucial to acupuncture efficacy, including the acupoints combination, stimulation frequency, and acupuncture retention duration, among others. Notably, acupuncture at acupoints is more effective than at non-acupoints (312, 313). Our analysis of the included studies reveals that the most commonly used acupoints for treating IS are *Baihui* (GV20), *Zusanli* (ST36), *Quchi* (LI11), *Dazhui* (GV14), *Shenting* (GV24), and *Shuigou* (GV26), which are consistent with recent systematic review outcomes (314, 315). Among these, *Baihui* (GV20), *Dazhui* (GV14), *Shenting* (GV24), and *Shuigou* (GV26) all belong to the Governor Vessel meridian; *Zusanli* (ST36) belongs to the Stomach Meridian. According to traditional Chinese medicine, the Governor Vessel “enters the brain”, while the Stomach Meridian “connects with the stomach.” This acupoint selection pattern indirectly supports the hypothesis that acupuncture exerts its effects through the brain-gut axis.

In terms of stimulation methods, parameter adjustment in manual acupuncture often relies on the doctor’s judgment and the patient’s subjective response, challenging the standardization of acupuncture therapy. On the contrary, since EA stimulation parameters are objectively quantified, the use of EA improves the reproducibility of the research. Interestingly, the included studies indicated that optimal EA parameters are not uniform. 5–20 Hz EA at *Baihui* (GV20)-*Dazhui* (GV14) on neurological deficits is the most significant (316); 15 and 30 Hz EA at *Zusanli* (ST36)-*Quchi* (LI11) best maintains the integrity of astrocytes (241); 2 Hz EA at *Shuigou* (GV26) or *Lianquan* (CV23) has the best recovery effect on swallowing function (317, 318); the analgesic effect of 15 Hz EA at *Baihui* (GV20)-*Zusanli* (ST36) is the most significant (319, 320). These seemingly contradictory parameters demonstrate the specificity of EA treatment: different frequencies activate different molecular pathways via different acupoint combinations to address specific pathological processes.

Needle retention time is another key factor affecting acupuncture efficacy. Although in clinical practice, the conventional acupuncture treatment lasts for 30 minutes, in terms of improving the permeability of the blood-brain barrier, the 8-minute EA at *Baihui* (GV20)-*Shuigou* (GV26) is more effective than the standard 30-minute protocol and causes less pain to rats (321). These findings suggest that the optimal acupuncture duration may vary depending on the specific therapeutic purpose, which is worthy of in-depth exploration in subsequent studies.

Unfortunately, the gap in key knowledge in this field still exists. Most evidence relies heavily on the rodent MCAO model, with limited data from other experimental models. Although the MCAO model simulates focal cerebral ischemia adequately, it cannot fully replicate the etiological heterogeneity, comorbidity background or chronic course of human IS. Another limitation is the fragmentation of existing evidence. We believe that acupuncture’s regulatory effects on the NEI network originate from peripheral stimulation and reach a climax in the response of specific target organs. This process consists of three basic stages: (1) local peripheral stimulation of acupoints, (2) signal transduction and transmission, and (3) integration and response of the CNS. However, the existing research mainly focuses on individual links or molecular targets. Comprehensive mechanism research that can fully connect these three links is still scarce. The further challenge lies in translating of basic research on acupuncture into clinical practice. This difficulty is related to variability in acupuncture protocols. For instance, in clinical practice, doctors usually perform acupuncture treatment during the recovery period of IS, but most mechanism studies are conducted in the acute or hyperacute phase, which limits their clinical applicability. In addition, there are significant differences among various studies in terms of acupoint selection and stimulation parameters, which weakens the comparability and potential for transformation of the results. Meanwhile, due to the lack of effective cross-species transformation methods for acupuncture stimulation parameters, the optimal stimulation parameters obtained in experimental studies have little guiding role in optimizing clinical treatment protocols. Furthermore, negative or conflicting findings are rarely reported in the current literature, suggesting potential publication bias. This imbalance may obscure a comprehensive understanding of acupuncture’s therapeutic limitations and context-dependent effects.

Future research should give priority to the following directions. The first step is to overcome the current fragmentation in this field. By adopting systems biology theories, integrating omics-based biomarkers, and strengthening causal verification for multi-dimensional observation, it will be possible to draw a clear and coherent biological landscape of the acupuncture therapy mechanism: from the site of needle insertion to the response of the entire organism. The next direction is to tackle the transformation barriers between basic research and clinical practice of acupuncture. To achieve this goal, it is essential to establish a framework of “clinical staging-biological response-optimal acupuncture parameters” to standardize acupuncture protocols. Meanwhile, artificial intelligence techniques, such as machine learning, can be employed to correlate various parameters of acupuncture with multimodal data from different stages and sequelae of IS, which will help to achieve personalized acupuncture treatment. Additionally, the potential role of NEI modulation in patient stratification or precision medicine will provide a clinical direction for the transformation of mechanism insights into individualized treatment. The above strategies will provide clear guidance on the following three questions: when to use acupuncture, how to use it, and why to use it. Collectively, these efforts will advance acupuncture therapy for IS toward greater standardization, precision, and internationalization.

## Supplementary material

Supplementary Table 1 Characteristics of the included studies.

## Glossary

2-AG: 2-arachidonoylglycerol
5-HT: 5-hydroxytryptamine
5-HT_2_A: 5-HT receptor 2A
A_1_R: adenosine A_1_ receptor
A20: TNF alpha-induced protein 3
AANAT: arylalkylamine N-acetyltransferase
ABIN1: A20-binding inhibitor of NF-κB activation 1
Abca1: ATP-binding cassette transporter A1
AC: adenylate cyclase
Ac: acetylation
AcbSh: nucleus accumbens shell
Ach: acetylcholine
ACTH: adrenocorticotropic hormone
AEA: anandamide
Akt: protein kinase B
AMPAR: α-amino-3-hydroxy-5-methyl-4-isoxazolepropionic acid receptor
Ang II: angiotensin II
ANXA1: annexin A1
Arg1: arginase 1
AT_1_R: Ang II receptor type 1
AT_2_R: Ang II receptor type 2
AUD: auditory cortex
Bax: BCL2-associated X protein
Bcl-2: B-cell lymphoma 2
BDNF: brain-derived neurotrophic factor
Bmal1: brain and muscle ARNT-like 1
CaMKII: Ca^2+^/calmodulin-dependent protein kinase II
cAMP: cyclic adenosine monophosphate
Caspase-1: cysteinyl aspartate specific proteinase-1
Caspase-3: cysteinyl aspartate specific proteinase-3
Caspase-8: cysteinyl aspartate specific proteinase-8
CbP: posterior lobe of cerebellum
CB_1_R: cannabinoid receptor type 1
CC: corpus callosum
CCL2: C-C motif chemokine ligand 2
CD: cluster of differentiation
Cdc42: cell division control protein 42
CDK: cyclin-dependent kinase
cADPR: cyclic ADP-ribose
CG: cingulate gyrus
ChAT: choline acetyltransferase
Clock: circadian locomotor output cycles kaput
CNS: central nervous system
CP: caudate putamen
CREB: cAMP response element binding protein
CX3CL1: C-X3-C motif chemokine ligand 1
CX3CR1: C-X3-C motif chemokine receptor 1
Cx43: connexin 43
CYLD: cylindromatosis
DAG: diacylglycerol
DRD2: dopamine receptor D2
DT: dorsal thalamus
EA: electroacupuncture
eNOS: endothelial nitric oxide synthase
EPO: erythropoietin
EpoR: EPO receptor
ER: estrogen receptor
ERK1/2: extracellular signal-regulated kinase 1/2
F-actin: filamentous actin
FADD: Fas-associated protein with death domain
Fizz1: found in inflammatory zone 1
FNDC5: fibronectin type III domain-containing protein 5
Foxp3: forkhead box P3
FPR: formyl peptide receptor
GABA: gamma-aminobutyric acid
GABA_A_R: GABA type A receptor
GABA_B_R: GABA type B receptor
GABAT: GABA transaminase
GAP-43: growth-associated protein 43
Gas7: growth arrest-specific 7
GFAP: glial fibrillary acidic protein
GLT-1: Glu transporter 1
Glu: glutamate
GluN2A: N-methyl-D-aspartate receptor subunit 2A
GluN2B: N-methyl-D-aspartate receptor subunit 2B
G_q_: Gq protein
Grm1a: metabotropic glutamate receptor 1a
GSDMD: gasdermin D
GSK-3: glycogen synthase kinase-3
GzmB: granzyme B
Hes1: hairy and enhancer of split 1
HIF-1α: hypoxia-inducible factor 1-alpha
HIP: hippocampus
HMGB1: high mobility group box 1
HO-1: heme oxygenase 1
HPA: axis hypothalamic-pituitary-adrenal axis
Iba1: ionized calcium-binding adapter molecule 1
IFN-γ: interferon-gamma
IGF-1: insulin-like growth factor-1
IκBα: inhibitor of kappa B alpha
IKKα: IκB kinase α
IKKβ: IκB kinase β
IL: interleukin
iNOS: inducible nitric oxide synthase
IP_3_: inositol trisphosphate
IPA: indole-3-propionic acid
IS: ischemic stroke
JAK2: Janus kinase 2
JNK: c-Jun N-terminal kinase
KCC2: K^+^-Cl^-^ cotransporter 2
KIF5b: kinesin family member 5B
L1cam: L1 cell adhesion molecule
LC3II/I: microtubule-associated protein 1A/1B-light chain 3
LIMK1: LIM domain kinase 1
LINGO-1: leucine-rich repeat and immunoglobulin domain-containing protein 1
lncRNA: long non-coding RNA
LPA: lysophosphatidic acid
LPS: lipopolysaccharide
m^6^A: N^6^-methyladenosine
M1: primary motor cortex
M1: microglia pro-inflammatory microglia
M2: microglia anti-inflammatory microglia
m-calpain: muscle calpain
mAChR: muscarinic acetylcholine receptor
MBP: myelin basic protein
MC: motor cortex
MCAO: middle cerebral artery occlusion
MCPIP1: monocyte chemotactic protein-induced protein 1
MCT1: monocarboxylate transporter 1
METTL3: methyltransferase-like 3
mGluR: metabotropic glutamate receptor
miR: microRNA
Miro1: mitochondrial Rho GTPase 1
MLC1: myosin light chain 1
MMP-9: matrix metalloproteinase-9
MPO: myeloperoxidase
MT: midbrain tegmentum
MT1: melatonin receptor 1
mtDNA: mitochondrial DNA
MTL: motilin
mTOR: mechanistic target of rapamycin
MY: medulla oblongata
MyD88: myeloid differentiation primary response 88
MYPT1: myosin phosphatase target subunit 1
NDRG2: n-myc downstream regulated gene 2
NEI: neuro-endocrine-immune
NeuroD1: neurogenic differentiation factor 1
NETs: neutrophil extracellular traps
NF-κB: nuclear factor-kappa B
NGF: nerve growth factor
NGL-3: netrin-G ligand-3
NgR: Nogo receptor
NK: cells natural killer cells
NKG2D: natural killer group 2D
NLRP3: NOD-like receptor family pyrin domain containing 3
NMDAR: N-methyl-D-aspartate receptor
Nogo-A: neurite outgrowth inhibitor A
Notch1: neurogenic locus notch homolog protein 1
NRF1: nuclear respiratory factor 1
Nrf2: nuclear factor erythroid 2-related factor 2
NTS: solitary nucleus
OMgp: oligodendrocyte-myelin glycoprotein
OTULIN: OTU deubiquitinase with linear linkage specificity
p21: cyclin-dependent kinase inhibitor 1A
p27: cyclin-dependent kinase inhibitor 1B
p38: p38 mitogen-activated protein kinase
p75^NTR^: p75 neurotrophin receptor
P2X7R: purinergic receptor P2X7
P2Y1R: purinergic receptor P2Y1
Parkin: parkin RBR E3 ubiquitin protein ligase
PBN: parabrachial nucleus
PC: parietal cortex
PCNA: proliferating cell nuclear antigen
PGC-1α: peroxisome proliferator-activated receptor gamma coactivator 1-alpha
PI3K: phosphatidylinositol 3-kinase
PINK1: PTEN-induced putative kinase 1
PIR: piriform cortex
PirB: paired immunoglobulin-like receptor B
PKA: protein kinase A
PKCϵ: protein kinase C epsilon
PL: prelimbic area
POA: preoptic area
PPARγ: peroxisome proliferator-activated receptor gamma
PRG5: plasticity-related gene 5
pro-BDNF: precursor of brain-derived neurotrophic factor
PSD-95: postsynaptic density protein 95
PT: pontine tegmentum
PTEN: phosphatase and tensin homolog
Rac1: Ras-related C3 botulinum toxin substrate 1
RAGE: receptor for advanced glycation end products
Rb: retinoblastoma protein
RhoA: Ras homolog family member A
ROCK2: Rho-associated coiled-coil containing protein kinase 2
ROS: reactive oxygen species
RSP: retrosplenial cortex
S100B: S100 calcium-binding protein B
S1PR2: sphingosine-1-phosphate receptor 2
S6: ribosomal protein S6
SC: superior colliculus
SCFAs: short-chain fatty acids
SERT: serotonin transporter
SN: substantia nigra
Sox2: SRY-box transcription factor 2
Src: Src tyrosine kinase
SSC: somatosensory cortex
STAT3: signal transducer and activator of transcription 3
STAT5: signal transducer and activator of transcription 5
STAT6: signal transducer and activator of transcription 6
ST2: growth stimulation-expressed gene 2
STR: striatum
SUMO: small ubiquitin-like modifier
SYN: synaptophysin
TAZ: tafazzin
TFAM: mitochondrial transcription factor A
TGF-β: transforming growth factor-beta
Th: T helper cells
TIMP: tissue inhibitor of metalloproteinase
TLR4: Toll-like receptor 4
TMAO: trimethylamine N-oxide
TNF-α: tumor necrosis factor-alpha
tPA: tissue-type plasminogen activator
TRAF6: TNF receptor associated factor 6
TRAK1: trafficking kinesin protein 1
TRADD: TNFRSF1A-associated via death domain
TREM2: triggering receptor expressed on myeloid cells 2
Tregs: regulatory T cells
TrkA: tropomyosin receptor kinase A
TrkB: tropomyosin receptor kinase B
TRPV1/4: transient receptor potential vanilloid 1/4
Ub: ubiquitination
UCP2: uncoupling protein 2
VEGF: vascular endothelial growth factor
VGLUT1: vesicular glutamate transporter 1
VIP: vasoactive intestinal peptide
VIS: visual cortex
Wnt1: Wnt family member 1
YAP: Yes1 associated transcriptional regulator
Ym1: chitinase 3 like 3
α7nAChR: α7 nicotinic acetylcholine receptor
γδ T cells: gamma delta T cells
