# Supplementary material for: Novel insights of acupuncture in ischemic stroke: orchestrating neuro-endocrine-immune network
Source: Front Immunol. 2026 Mar 19;17:1772371. doi: 10.3389/fimmu.2026.1772371 (PMC13043432; doi:10.3389/fimmu.2026.1772371)
Supplement: Supplementary Table 1 — Characteristics of the included studies. [file Table1.docx]

**Table S1.** Characteristics of the included studies.

| **Models** | **Acupuncture Methods** | **Acupoints** | **Acupuncture Parameters** | **Modulatory molecules (Acupuncture vs. Model)** | **Major Targeting Pathophysiology** | **Phase** | **Ref** |
| --- | --- | --- | --- | --- | --- | --- | --- |
| pMCAO in SD rats | MA | GV26 | Started at 2 h post-modeling, 30 min, once daily for 3 d | STR: Glu↓, GABA↑ | Glu, GABA | Acute phase | [29] |
| pMCAO in SD rats | EA | TE5, LI11, GB34, ST36 | Started at 5 d post-modeling, 2 Hz sparse waves, 25 min, once daily for 7 d | Ischemic penumbra cortex: Glu↓, GABA↑, Grm1a mRNA↓, Gabbr1 mRNA↑ | Glu, GABA | Subacute phase | [30] |
| 120 min tMCAO in SD rats | EA | GV20, GV24 | Started at 2 h post-modeling, 20 Hz, 30 min, once daily for 7 d | CA1: Glu↓, Ca2+↓, GluN2A↑, GluN2B↓ | Glu | Subacute phase | [34] |
| 90 min tMCAO in SD rats | EP | GV20, PC6, SP6 | 2/15 Hz, 1 mA, 20 min, once daily for 5 d, with modeling conducted at 2 h post-treatment | CA1: GluN2B↓, m-calpain↓, p-p38↓, p-p38/p38↓ | Glu | Hyperacute phase | [35] |
| 120 min tMCAO in SD rats | EP | GV20, GV14 | 2/15 Hz, 30 min, once daily for 5 d, with modeling conducted at 24 h post-treatment | STR: Glu↓, GLT-1↑ | Glu | Hyperacute phase | [38] |
| 120 min tMCAO in SD rats | EA | GV20, GV26 | Started at 15 min post-modeling, 3.58-6.25 Hz, 1.4-2.0 mA, 30 min stimulation, with a 10 min pause, then for 30 min again | Ischemic penumbra cortex: GABA↑ | GABA | Acute phase | [47] |
| pMCAO in SD rats | MA | EX-B2 | Started post-modeling, 30 s clockwise rotation at 2-3 rotations/s, once daily for 7 d | STR and spinal cord: β-endorphin↑, GABAARγ2↑, GABAB2↑ | GABA, opioid peptides | Subacute phase | [48] |
| 120 min tMCAO in SD rats | EA | LI4, TE5, GB34, ST36 | Started at 1 d post-modeling, 1/20 Hz, 30 min, once daily for 7 d | Ischemic penumbra tissues: GABAB1 mRNA and protein↑, GABAB2 mRNA and protein↑, AC↑, cAMP↑, PKA↑, CREB mRNA and protein↑ | GABA | Acute phase | [49] |
| pMCAO in SD rats | MA | GB34 | Started at 3 d post-modeling, bidirectional needle manipulation, ankle mobilization, and needle retention within a 30 min session, once daily for 7 d | Ischemic penumbra: GABA↑, GABAT↓ | GABA | Subacute phase | [50] |
| pMCAO in SD rats | MA | GB34 | Started at 3 d post-modeling, bidirectional needle manipulation, ankle mobilization, and needle retention within a 30 min session, once daily for 7 d | Ischemic penumbra: KCC2 mRNA and protein↑, GABAARγ2 mRNA and protein↑ | GABA | Subacute phase | [51] |
| pMCAO in SD rats | MA | GB34 | Started at 3 d post-modeling, bidirectional needle manipulation, ankle mobilization, and needle retention within a 30 min session, once daily for 7 d | Brainstem: GABA↑, KCC2 mRNA and protein↑, GABAARγ2 mRNA and protein↑ | GABA | Subacute phase | [52] |
| pMCAO in SD rats | MA | GB34 | Started at 3 d post-modeling, bidirectional needle manipulation, ankle mobilization, and needle retention within a 30 min session, once daily for 7 d | lumbar spine: GABA↑, KCC2 mRNA and protein↑,GABAARγ2 mRNA and protein↑ | GABA | Subacute phase | [53] |
| 120 min tMCAO in SD rats and C57BL/6 mice | EP | GV20 | 2/15 Hz, 1 mA, 30 min, once daily for 3 d, with modeling conducted at 2 h post-treatment | Ischemic penumbra: AEA↑, 2-AG↑, CB1R mRNA and protein↑ | Endocannabinoid | Acute phase | [56] |
| 120 min tMCAO in SD rats | EP | GV20 | 2/15 Hz, 1 mA, 30 min, once daily for 5 d, with modeling conducted at 1 d post-treatment | Ischemic hemisphere: CB1R↑, p-ERK1/2↑ | Endocannabinoid | Hyperacute phase | [57] |
| 120 min tMCAO in SD rats | EP | GV20 | 2/15 Hz, 1 mA, 30 min, with modeling at 2.5 h post-treatment | Ischemic penumbra: CB1R↑, p-GSK-3β(Ser-9)/GSK-3β↑, Bax/Bcl-2↓ | Endocannabinoid | Hyperacute and acute phase | [58] |
| 120 min tMCAO in SD rats | EP | GV20 | 2/15 Hz, 1 mA, 30 min, with modeling conducted at 2 h post-treatment | Ischemic penumbra: p-STAT3(Ser727)/STAT3↑, Caspase-3↓, Bax/Bcl-2↓ | Endocannabinoid | Acute phase | [59] |
| 120 min tMCAO in SD rats | EP | GV20 | 2/15 Hz, 1 mA, 30 min, with modeling conducted at 2 h post-treatment | Ischemic penumbra: CB1R↑, PKCε membrane/cytosol↑, Bcl-2↑, Bax↓ | Endocannabinoid | Acute phase | [60] |
| 60 min tMCAO in C57BL/6 mice | EP | GV20 | 2/15 Hz, 1 mA, 30 min, with modeling conducted at 2 h post-treatment | Ischemic penumbra: CB1R↑, PGC-1α↑, NRF1↑, TFAM↑, mtDNA↑, cyto C↓, COX IV↑, mPTP opening↓, MMP↑ | Endocannabinoid | Hyperacute phase | [61] |
| 90 min tMCAO in C57BL/6 mice | EP | GV20 | 2/15 Hz, 1 mA, 30 min, with modeling conducted at 2 h post-treatment | CA1: p-GSK-3β/GSK-3β↑ | Adenosine | Hyperacute phase | [67] |
| 120 min tMCAO in SD rats | EP | GV20 | 2/15 Hz disperse waves, 1 mA, 30 min, with modeling conducted at 2 h post-treatment | HIP: Bcl-2/Bax↑ | Adenosine | Hyperacute phase | [68] |
| 90 min tMCAO in SD rats | EA | GB20 | Started at 1 d post-modeling, 2 Hz continuous waves, 3 mA, 20 min stimulation,with a 10 min pause, then for 20 min again, once daily for 7 d | STR: GAP-43^+^ tyrosine hydroxylase^+^ cells↑ | Dopamine | Acute phase | [72] |
| 120 min tMCAO in SD rats | EP | GV20 | 2/15 Hz, 1 mA, 30 min, once daily for 5 d, with modeling conducted at 1 d post-treatment | STR: Met-enkephalin↑ | Opioid peptides | Hyperacute phase | [74] |
| pMCAO in SD rats | EA | GV20, ST36 | Started at 1 d post-modeling, 2/10 Hz, 30 min, once daily for 14 d | Hypothalamus: 5-HT↑, SERT↑, 5-HT2A↓ | 5-HT | Subacute phase | [75] |
| 120 min tMCAO in SD rats | EA | LI11, ST36 | Started at 1 d post-modeling, 2/10 Hz, 30 min, once daily for 7 d | Ischemic penumbra: BDNF mRNA and protein↑, p-TrkB/TrkB↑, PSD-95 mRNA and protein↑, Synapsin-1 mRNA and protein↑, Nogo-A mRNA and protein↓, NgR mRNA and protein↓ | Synaptic plasticity | Acute phase | [79] |
| 120 min tMCAO in SD rats | EA | GV20, GB7 | 3 Hz bipolar waves, twice daily for 14 d | Ischemic hemisphere: BDNF↑, TrkB↑ | Synaptic plasticity | Subacute phase | [80] |
| pMCAO in SD rats | EA | LI4, LR3 | Started at 14 d post-modeling, 2-20 Hz disperse-dense waves, 30 min, once daily for 21 d | HIP: BDNF↑, TrkB↑ | Synaptic plasticity | Chronic phase | [81] |
| 90 min tMCAO in SD rats | EA | GV20, GV24 | Started at 1 d post-modeling, 2/10 Hz, 1-3 mA, 30 min, once daily for 7 d | HIP: BDNF↑, TrkB↑, PSD-95↑ | Synaptic plasticity | Acute phase | [82] |
| 90 min tMCAO in SD rats | EA | GV20, GV29, CV12, CV4 | Started at 14 d post-modeling, 2 Hz, 1 mA, 30 min, once daily for 24 d | PFC: tPA↑, pro-BDNF↓, BDNF↑, TrkB↑; Serum: BDNF↑, pro-BDNF↓, BDNF/pro-BDNF↑ | Synaptic plasticity | Chronic phase | [83] |
| 120 min tMCAO in SD rats | EA | GV20, GV29 | Started at 1 d post-modeling, 2 Hz, 1 mA, 10 min, once daily for 14 d | PFC and HIP: BDNF↑, TrkB↑, AMPAR↑, GluN1↑, GABAAR↑, CaMKII↑, PSD-95↑ | Synaptic plasticity | Subacute phase | [84] |
| 60 min tMCAO in C57BL/6 mice | EA | GV24, GB13 | Started at 1 week post-modeling, 2 Hz, 0.3 mA, 15 min, 5 sessions/week for 21 d | S1 and PFC: c-fos↑; PFC: NGL-3 mRNA and protein↑,VGLUT1↑, VGLUT1^+^ MAP2^+^ cells↑ | Synaptic plasticity | Chronic phase | [85] |
| 120 min tMCAO in SD rats | EA | GV20, GV24 | Started at 4 h post-modeling, 1-20 Hz disperse waves, 30min, once daily for 7 d | CA1: RhoA↓, Cdc42↑, Rac1↑, F-actin↑ | Synaptic plasticity | Acute phase | [89] |
| 90 min tMCAO in SD rats | EA | GV20, GV24 | Started at 1 d post-modeling, 1-20 Hz dilatational waves, 6 V, 0.2 mA, 30 min, once daily for 14 d | CA1: miR-134↓, t-LIMK1↑, p-LIMK1↑ | Synaptic plasticity | Subacute phase | [90] |
| pMCAO in SD rats | EA | GV20, ST36 | Started at 1 d post-modeling, 2/10 Hz dense-sparse waves, 1 mA, 30 min, once daily for 14 d | White matter in internal capsule: Nogo-A↓, NgR↓, MBP↑ | Synaptic plasticity | Subacute phase | [95] |
| 120 min tMCAO in SD rats | EA | GV20, ST36 | Started post-modeling, 2 Hz continuous waves, 1 mA, 30 min, once daily for 7 d | Ischemic penumbra: Nogo-A↓, OMgp↓, NgR mRNA and protein↓, RhoA↓, ROCK2 mRNA and protein↓, MLC1 mRNA and protein↓, MYPT1↓, GAP-43↑, BDNF↑ | Synaptic plasticity | Acute phase | [96] |
| pMCAO in SD rats | EA | LI11, ST36 | Started at 1 d post-modeling, 1-20 Hz dense-disperse waves, 30 min, once daily for 14 d | Ischemic penumbra: Nogo-A mRNA and protein↓, NgR mRNA and protein↓, RhoA mRNA and protein↓, ROCK mRNA and protein↓, GAP-43 mRNA and protein↑ | Synaptic plasticity | Acute and subacute phase | [97] |
| 120 min tMCAO in SD rats | MA | GV20, LI11, ST36 | Started at 1 d post-modeling, 30 min, once daily for 14 d | Cortex: Nogo-A↓, NgR↓, p75NTR↓, LINGO-1↓ | Synaptic plasticity | Subacute phase | [98] |
| 90 min tMCAO in SD rats | EA | GV20 | Started at 1 d post-modeling, 2/10 Hz dense-disperse waves, 1-2 mA, 30 min, once daily for 5 d | Ischemic penumbra: miR-132↑, Sox2↓ | Synaptic plasticity | Acute phase | [99] |
| 120 min tMCAO in SD rats | EA | GV20 | Started at 2 h post-modeling, 2/10 Hz dense-disperse waves, 1-2 mA, 30 min, once daily with 5 sessions/week for 28 d | Ischemic penumbra: miR-181b↑, PirB mRNA and protein↓, RhoA↓, GAP-43↑ | Synaptic plasticity | Chronic phase | [100] |
| pMCAO in SD rats | EA | PC6, ST36 | Started at 1 d post-modeling, 2 Hz dense waves, 1 mA, 20 min, once daily for 14 d | Cortex: p-PTEN/t-PTEN↓, p-Akt/t-Akt↑, p-mTOR/t-mTOR↑, p-S6/t-S6↑, p-S6↑, GAP-43↑, SYN↑ | Synaptic plasticity | Subacute phase | [101] |
| 120 min tMCAO in SD rats | EA | LI11, ST36 | Started at 1 d post-modeling, 1-20 Hz dense-disperse waves, 30 min, once daily for 21 d | SVZ: BrdU↑, BrdU^+^ GFAP^+^ cells↑; STR: BrdU^+^ NeuN^+^ cells↑ | Neurogenesis | Acute, subacute, and chronic phase | [111] |
| pMCAO in SD rats | EA | GV20, GV26 | Started at 3 d post-modeling, 4-20 Hz disperse waves, 2-4 mV, 1-2 mA, 15 min, once daily for 21 d | DG: 3, 7, 14 d BrdU^+^/GFAP^+^ cells↑, Notch1 mRNA↑, Hes1 mRNA↑; 21d BrdU^+^ NeuN^+^ cells↑ | Neurogenesis | Acute, subacute, and chronic phase | [113] |
| 120 min tMCAO in SD rats | EA | LI11, ST36 | Started at 1 d post-modeling, 1 or 20 Hz, 0.01 mA, 30 min, once daily for 7 d | DG and CA1: Nestin↑, Notch1 mRNA and protein↑, Hes1 mRNA↑, p21↓, p27 mRNA and protein↓, Cyclin D1 mRNA and protein↑, CDK4 mRNA and protein↑, p-Rb mRNA and protein↑; Serum: BDNF↑, GDNF↑ | Neurogenesis | Acute phase | [114] |
| 120 min tMCAO in SD rats | EA | LI11, ST36 | Started at 2 h post-modeling, 2/15 Hz sparse-dense waves, 1 mA, 20 min, once daily for 3 d | Ischemic penumbra cortex and STR: Cyclin D1↑, CDK4↑, p-Rb↑, BDNF↑, GFAP^+^ vimentin^+^ cells↑, GFAP^+^ Nestin^+^ cells↑, GFAP^+^ BrdU^+^ cells↑ | Neurogenesis | Acute phase | [115] |
| 90 min tMCAO in SD rats | EA | LI11, ST36 | Started at 1 d post-modeling, 20 Hz continuous waves, 1 mA, 30 min, once daily for 7 d | HIP and SVZ: Notch1↑, miR-223↑, PTEN↓, Nestin↑ | Neurogenesis | Acute phase | [116] |
| 120 min tMCAO in SD rats | EA | GV20, GV14 | Started post-modeling, 5/20 Hz disperse-dense waves, 2-4 mA, 30 min, once daily for 14 d | DG: 1, 7, 14 d BrdU^+^ Nestin^+^ cells↑, BrdU^+^ DCX^+^ cells↑; 7 d PRG5↑, Nogo-A↓, LPA↓, RhoA↓ | Neurogenesis | Acute and subacute phase | [117] |
| 40 min tMCAO in C57BL/6 mice | EA | GV20, GV14 | Started at 5 d post-modeling, 2 Hz, 2 V, 20 min, once daily for 10 d | HIP and SVZ: 14 d BrdU↑, BDNF mRNA and protein↑, VEGF mRNA and protein↑, BrdU^+^ DCX^+^ cells↑, BrdU^+^ NeuN^+^ cells↑, BrdU^+^ mBDNF^+^ cells↑, BrdU^+^ VEGF^+^ cells↑, BrdU^+^ p-PI3K^+^ cells↑; 47 d BrdU^+^ NeuN^+^ cells↑, BrdU^+^ GFAP^+^ cells↑ | Neurogenesis | Subacute and chronic phase | [118] |
| 120 min tMCAO in SD rats | EA | LI11, ST36 | Started at 1 h post-modeling, 1-20 Hz dilatational waves, 6 V, 2 mA, 30 min, once daily for 3 d | Ischemic penumbra cortex: p-ERK1/2↑, PCNA↑, p21 mRNA and protein↓, p27 mRNA and protein↓, Cyclin D1 mRNA and protein↑, Cyclin E mRNA and protein↑, CDK2 mRNA and protein↑, CDK4 mRNA and protein↑ | Neurogenesis | Acute phase | [119] |
| 120 min tMCAO in SD rats | EA | LI11, ST36 | Started at 1 d post-modeling, 1 or 20 Hz dense-disperse waves, 30min, once daily for 3 d | Ischemic penumbra: Wnt1 mRNA and protein↑, β-catenin mRNA and protein↑, GSK-3 mRNA and protein↓, GFAP↑, Nestin^+^ GFAP^+^ cells↑ | Neurogenesis | Acute phase | [121] |
| 90 min tMCAO in SD rats | EA | LI11, ST36 | Started post-modeling, 1/20 Hz, 1 mA, 30 min, once daily for 21 d | STR: miR-125b↑, miR-146b↑, miR-30a↑, miR-1949↓; SVZ: NeuroD1↑, NeuroD1^+^ DCX^+^ cells↑; STR and SVZ: BrdU^+^ NeuN^+^ cells↑ | Neurogenesis | Chronic phase | [122] |
| pMCAO in SD rats | EA | GV20, ST36 | Started at 7 d post-modeling, 2 Hz, 2 V, 1 mA, 30 min, once daily for 21 d | PFC: Gas7↑, NGF↑ | Neurogenesis | Chronic phase | [123] |
| 120 min tMCAO in SD rats | EA | LI11, ST36 | Started at 1 d post-modeling, 1/20 Hz dense-disperse waves, 6 V, 2 mA, 30min, once daily for 7 d | ReHo in the left MC, left STR, right AUD, right RIP, bilateral DT, bilateral HIP, and bilateral SSC↑ | Functional brain connectivity | Acute phase | [128] |
| 120 min tMCAO in SD rats | EA | LI11, ST36 | Started at 1 d post-modeling, 2/15 Hz, 30 min, once daily for 7 d | ReHo in the left posterior dorsal HIP, left CC, left SSC, right anterior dorsal HIP, right subiculum HIP, and right DT↑; ALFF in the left caudate putamen and left anterodorsal HIP↑ | Functional brain connectivity | Acute phase | [129] |
| 90 min tMCAO in SD rats | EA | LI11, ST36 | Started at 1 d post-modeling, 2/20 Hz dense-disperse waves, 30 min, once daily for 14 d | FC of the left MC with the left CbP, left MC, left RIP, left SSC, left STR, left VIS, left CG, left preoptic area, left parietal cortex, right posterior lobe of cerebellum, bilateral medulla oblongata, and bilateral pontine tegmentum↑ | Functional brain connectivity | Subacute phase | [130] |
| 120 min tMCAO in SD rats | EA | LI11, ST36 | Started at 1 d post-modeling, 1/20 Hz dense-disperse waves, 6 V, 2 mA, 30min, once daily for 7 d | FC of the left STR with the left RSP, left parietal cortex, left CG, left superior colliculus, right cerebellum, bilateral SSC, bilateral MC, bilateral AUD, bilateral VIS, and bilateral HIP↑ | Functional brain connectivity | Acute phase | [131] |
| 120 min tMCAO in SD rats | EA | LI11, ST36 | Started at 1 d post-modeling, 2/15 Hz, 30 min, once daily for 7 d | FC of the Sensorimotor Network (right CC), Interosseous Network (left VIS), central executive network (right AcbSh and SSC), and salience network (right MC)↑ | Functional brain connectivity | Acute phase | [132] |
| 120 min tMCAO in SD rats | EA | LI11, ST36 | Started at 1 d post-modeling, 2/15 Hz, 0.1 mA, 20 min, once daily for 7 d | BC of the right HIP ventral↑, BC of the right substantia nigra↓; DC of the left AcbSh↓ | Functional brain connectivity | Acute phase | [133] |
| 120 min tMCAO in SD rats | EA | LI11, ST36 | Started at 1 d post-modeling, 2/20 Hz dense-disperse waves, 30 min, once daily for 7 d | glucose metabolism in the caudate putamen, MC and SSC↑ | Functional brain connectivity | Acute phase | [134] |
| 90 min tMCAO in SD rats | EA | GV20, GV24 | Started at 1 d post-modeling, 1-2Hz dilatational waves, 2 mA, 30 min, once daily for 14 d | ALFF in the left AUD, left RSP, left DT, left HIP, right CG, right prelimbic area, bilateral MC, and bilateral SSC↑ | Functional brain connectivity | Subacute phase | [135] |
| 90 min tMCAO in SD rats | EA | GV20, GV24 | Started at 1 d post-modeling, 1/20 Hz dense-disperse waves, 6 V, 0.2 mA, 30 min, once daily for 14 d | FC of the left RSP with the left HIP, right midbrain tegmentum, right VIS, bilateral RSP, and bilateral CG↑ | Functional brain connectivity | Subacute phase | [136] |
| 120 min tMCAO in SD rats | EA | GV20, GV24 | 1-20 Hz dilatational waves, 6 V, 1 mA, 30 min, once daily for 7 d | HIP and PFC: N-acetylaspartate↑, Choline↑ | Functional brain connectivity | Acute phase | [137] |
| M1 focal PT model in C57BL/6 mice | EA | CV23 | Started at 1 d post-modeling, 2 Hz continuous waves, 1 mA, 15 min, once daily for 1 or 3 d | M1 (Non-infarction area): local field potential energy↑, pyramidal cell (spike counts↑, peak value↑), total neuronal spike counts↑, blood perfusion↑; Hypoglossal nerve: spike counts↑, motor conduction velocity↑, latency of evoked electromyography↓; Swallowing muscle: EMG amplitude↑; Serum: SP↑ | Functional brain connectivity | Hyperacute and acute phase | [138] |
| M1 focal PT model in C57BL/6 mice | EA | CV23 | Started at 1 d post-modeling, 2 Hz continuous waves, 1 mA, 15 min | Contralateral M1: perineuronal nets↓, c-Fos↑, neuronal spike firing↑; Excitatory Neurons: frequency of sEPSCs ↑; GABAergic Neurons: frequency of sEPSCs ↑; Swallowing muscle: EMG amplitude↑ | Functional brain connectivity | Hyperacute phase | [139] |
| M1 focal PT model in C57BL/6 mice | EA | CV23 | Started at 1 d post-modeling, 2 Hz intermittent pulse, 1 mA, 15 min | Nucleus tractus solitarius: c-Fos↑, excitatory neurons (CaMKIIα) activation↑, pyramidal neurons firing rate↑, interneurons firing rate↑; Swallowing muscle: EMG amplitude↑, pharyngeal pressure↑ | Functional brain connectivity | Hyperacute phase | [140] |
| M1 focal PT model in C57BL/6 mice | EA | CV23 | Started at 1 d post-modeling, 2 Hz intermittent pulse, 1 mA, 15 min | Excitatory neurons in the contralateral M1 layer 5: c-Fos↑, somatic Ca²+transients↑, population neuronal activity↑; Parabrachial nucleus: c-Fos↑; Nucleus tractus solitarii: c-Fos↑, neuronal firing rate↑; Mylohyoid muscle: EMG amplitude and frequency↑, pharyngeal pressure↑ | Functional brain connectivity | Hyperacute phase | [141] |
| 120 min tMCAO in SD rats | EA | GV20, ST36 | Started at 30 min post-modeling, 2-100 Hz dilatational waves, 2 mA, 20 min, once daily for 2 d | Serum: ACTH↓, HSP70↓ | HPA axis | Acute phase | [146] |
| pMCAO in Wistar rats | EA | GV26 | Started post-modeling, 15 Hz, 1 mA, 20 min | Brain tissues: 1-3 h Ang II↓, 12-24 h Ang II↑, AT1R↓, AT2R↑, Gq↓, IP3↓, DAG↓, CaM↓ | Angiotensin | Hyperacute phase | [151] |
| 120 min tMCAO in SD rats | EA | GV20, ST36 | Started at 2 h post-modeling and again at 2 h before euthanasia, 2-100 Hz, 2 mA, 20 min | Ischemic penumbra: EPO mRNA and protein↑, EpoR mRNA and protein↑, JAK2 mRNA and protein↑, p-JAK2↑, p-JAK2/JAK2↑, STAT3 mRNA and protein↑, p-STAT3↑, p-STAT3/STAT3↑ | EPO | Hyperacute phase | [160] |
| pMCAO in SD rats | EA | GV20, GV14 | Started at 1 h post-modeling, 4/20 Hz, 1-2mA, 30 min, once daily for 3 d | Ischemic cortex: HIF-1α mRNA and protein↑, EPO mRNA and protein↑, p-JAK2↑, p-STAT5↑, HSP70 mRNA and protein↑, Bcl-2 mRNA and protein↑, Bax mRNA and protein↓ | EPO | Acute phase | [161] |
| 90 min tMCAO in Wistar rats | EA | GV26 | Started at 30 min before modeling and at 3, 12, and 24 h after reperfusion, 15 Hz, 1 mA, 20 min, once daily for 7 d | Brain tissues: EPO↑, p-Src↑, VEGF↑, CD34↑ | EPO | Acute phase | [162] |
| tMCAO in SD rats | EP | GV20 | 2/15 Hz, 30 min, once daily for 7 d, with modeling conducted at 2 d post-treatment | STR: p-ERα (Ser118)↑, ERβ↑; Ischemic penumbra: Bcl-2↑, cleaved Caspase-3↓ | Estrogen | Subacute phase | [165] |
| 90 min tMCAO in SD rats | EA | LI11, ST36 | 2-10 Hz intermittent waves, 1-2mA, 90 min, once daily for 3 d | Serum: Irisin↑; Ischemic penumbra cortex: FNDC5↑ | Irisin | Acute phase | [170] |
| 90 min tMCAO in SD rats | EA | ST36 | Started at 1 d post-modeling, 3/15 Hz dense-disperse waves, 30 min, once daily for 7 d | Muscle tissue: FNDC5↑, Irisin↑; Serum: Irisin↑; Ischemic penumbra cortex: FNDC5↑, BDNF↑, VEGF↑, p-Akt/Akt↑, p-eNOS/eNOS↑ | Irisin | Acute phase | [171] |
| 90 min tMCAO in SD rats | EA | GV20, GV24 | Started at 1 d post-modeling, 4 Hz sparse waves and 20 Hz dense waves, 2 V, 0.5 mA, 30 min, once daily for 7 d | Serum: Melatonin↑; Pineal gland: AANAT mRNA and protein↑; HIP: LC3II/I↑, PINK1↑, Parkin↑, ROS↓, NLRP3↓, ASC↓, Caspase-1↓, IL-1β↓, IL-18↓; CA1: Beclin-1^+^ NeuN^+^ cells↑, Iba1↓ | Melatonin | Acute phase | [175] |
| pMCAO in SD rats | EA | GV20, BL23 | Started at 2 h post-modeling, 2 Hz, 1-2 mA, 15 min, once daily for 3 d | Ischemia tissues: Bmal1/Clock↑, Bcl-2/Bax↑, Caspase-3↓; Serum: IL-1β↓, IL-6↓, IL-8↓ | Circadian rhythms | Acute phase | [179] |
| 120 min tMCAO in SD rats | EA | GV20, LI4, LR3 | Started post-modeling, 2/20 Hz, 1 mA, 30 min, once daily for 7 d | Ischemic penumbra cortex: Iba1↓, OTULIN mRNA and protein↑, p-IκBα/IκBα↓, nuclear/cytoplasm NF-κB p65↓, TNF-α↓, IL-1β↓, IL-6↓, Iba1^+^ OTULIN^+^ cells↑, NeuN^+^ OTULIN^+^ cells↑ | Microglia | Acute phase | [184] |
| 60 min tMCAO in C57BL/6 mice | EA | CV24, GV26 | Treatment was applied for 30 min before and after reperfusion using dense-sparse waves (16 Hz for 1.5 s alternating with 4 Hz for 1.5 s). Stimulation voltage began at 1 V and increased by 1 V every 10 min to a final 3 V | Cortex: NeuN↑, Iba1↓, ANXA1 mRNA and protein↑, FPR mRNA and protein↑, BDNF mRNA and protein↑, TNF-α mRNA and protein↓, IL-1β mRNA and protein↓, iNOS mRNA↓, Arg1 mRNA and protein↑ | Microglia | Hyperacute phase | [185] |
| 120 min tMCAO in SD rats | EA | GV20, GV24 | Started post-modeling, 0.05 Hz disperse waves, 6 V, 30 min, once daily for 7 d | CA1 in the neurons: IκBα↑, nucleus/cytoplasm NF-κB p65↓; CA1: TNF-α↓, IL-1β↓, IL-6↓, CD45↓ | Microglia | Acute phase | [188] |
| pMCAO in SD rats | EA | GV20, GV14 | Started post-modeling, sparse-dense waves, 1-2 mA, 30 min, once daily for 3 d | Ischemic penumbra: Iba1↓, NF-κB p65↓, TNF-α↓, IL-1β↓, CD11b↓ | Microglia | Acute phase | [189] |
| 120 min tMCAO in SD rats | EA | LI11, ST36 | Started at 1 d post-modeling, 1-20 Hz dilatational waves, 6 V, 0.2 mA, 30 min, once daily for 3 d | Sensorimotor cortex: Iba1↓, CD68↓, MyD88↓, p38↓, IκBα↑, NF-κB p65↓, nucleus NF-κB p65↓, TNF-α↓, IL-1β↓, IL-6↓; Serum: TNF-α↓, IL-1β↓, IL-6↓ | Microglia | Acute phase | [191] |
| pMCAO in Wistar rats | EA | PC6 or LI11 or SP8 | 2/15 Hz sparse-dense waves, 1 mA, 30 min, once daily for 5 d | Ischemic tissues: (P6) TLR4↓, TRAF6↓, IKKβ↓, NF-κB p65↓, IL-1β↓; (LI11) TLR4↓, TRAF6↓, IKKβ↓, NF-κB p65↓, TNF-α↓, IL-6↓; (SP8) TRAF6↓, IKKβ↓, NF-κB p65↓, IL-1β↓ | Microglia | Acute phase | [192] |
| 120 min tMCAO in SD rats | EA | LI11, ST36 | Started at 2 h post-modeling, 1/20 Hz, 0.01 mA | Ischemic cortex: TLR4 mRNA and protein↓, p-IκBα↓, NF-κB p65 mRNA and protein↓, TNF-α↓, IL-1β↓, IL-6↓ | Microglia | Hyperacute phase | [193] |
| 120 min tMCAO in SD rats | EA | GV20, PC6 | Started post-modeling, 20 Hz dense-disperse waves, 1-2 mA, 20 min, twice daily with 6 sessions/week for 21 d | HIP: TLR4↓, MyD88↓, NF-κB↓, NLRP3 mRNA and protein↓, ASC mRNA↓, Caspase-1 mRNA↓, TLR4^+^ Iba1^+^ cells↓, NLRP3^+^ Iba1^+^ cells↓; Serum: TNF-α↓, IL-1β↓, IL-6↓, IL-18↓ | Microglia | Chronic phase | [194] |
| 120 min tMCAO in SD rats | EP | GV20 | 2/15 Hz sparse-dense waves, 1 mA, 30 min, once daily with 6 sessions/week for 14 d, with modeling conducted at 48 h post-treatment | Brain tissues: Iba1↓, TLR4↓, NF-κB p65↓, TXNIP↓, TRX1↑, NLRP3↓, IL-1β↓, IL-18↓, iNOS↓, IFN-γ↓, Arg1↑, TGF-β1↑, IL-4↑, Iba1^+^ CD16^+^ cells↓, Iba1^+^ CD206^+^ cells↑, Iba1^+^ TLR4^+^ cells↓, Iba1^+^ NLRP3^+^ cells↓ | Microglia | Hyperacute phase | [195] |
| 60 min tMCAO in C57BL/6 mice | EA | ST36 | Started at 1 d post-modeling, 4/20 Hz disperse-dense waves, 4 V, 30 min, once daily for 4 weeks | HIP: TLR4↓, p-p38↓, p-NF-κB↓, NLRP3↓, TNF-α↓, IL-1β↓, IL-6↓; DG: Iba1↓, Iba1^+^ CD86^+^ cells↓, Iba1^+^ NLRP3^+^ cells↓, Edu^+^ NeuN^+^ cells↑, DCX↑; Peripheral blood: CD4^+^/CD8^+^ T cells↑, CD3^-^CD49b/CD45^+^CD49b^+^ cells↑ | Microglia | Chronic phase | [196] |
| 120 min tMCAO in SD rats | EA | GV20, LI4, LR3 | Started post-modeling, 2/20 Hz, 1 mA, 30 min, once daily for 3 d | Ischemic penumbra: TREM2 mRNA and protein↑, p-PI3K/PI3K↑, p-Akt/Akt↑, p-NF-κB p65/NF-κB p65↓, TNF-α↓, IL-1β mRNA↓, IL-6 mRNA↓, Arg1 mRNA↑, IL-10 mRNA↑, TREM2^+^ Iba1^+^ cells↑ | Microglia | Acute phase | [197] |
| pMCAO in SD rats | EA | GV20, ST36 | Started at 1 d post-modeling, 2/10 Hz alternating waves, 1 mA, 30 min, once daily for 14 d | CC: HMGB1↓, RAGE↓ | Microglia | Subacute phase | [199] |
| pMCAO in SD rats | EA | GV20, ST36 | Started at 1 d post-modeling, 2 Hz, 1 mA, 30 min, once daily for 14 d | STR: HMGB1↓, RAGE↓, p-JNK↓ | Microglia | Subacute phase | [200] |
| pMCAO in SD rats | EA | GV20, ST36 | Started at 1 d post-modeling, 2 Hz, 1 mA, 30 min, once daily for 14 d | M1: HMGB1↓, RAGE↓ | Microglia | Subacute phase | [201] |
| 120 min tMCAO in SD rats | EP | GV20, BL23, SP6 | Stimulation (2/100 Hz, 1 mA) applied in four 10 min cycles interspersed with 5 min retention periods (1 h total) | HIP: TRPV1↓, p-p38/p38↓, MDA↓, GSH↑, SOD↑, cyto C↓, TNF-α↓, IL-1β↓ | Microglia | Hyperacute phase | [203] |
| 60 min tMCAO in SD rats | EA | GV20, PC6, Ki1 | Started at 1 h post-modeling, 1-20 Hz dilatational waves, 6 V, 2 mA, 30 min, once daily for 7 d | HIP: Iba1↓, CD86↓, CD206↑, p-p38/p38↓, TNF-α↓, iNOS↓, IL-6↓, Arg1↑, IL-4↑, IL-10↑ | Microglia | Acute phase | [204] |
| 30 min tMCAO in C57BL/6 mice | EA | CV24, GV26 | Started post-modeling, 4/20 Hz density waves, 1-3 V, 1-3 mA, 20 min, once daily for 3 d | Ischemic cortex: Iba1↓, TRPV4 mRNA and protein↓, TNF-α mRNA↓, IL-1β mRNA↓, IL-6 mRNA↓, iNOS↓, CCL2 mRNA↓ | Microglia | Acute phase | [205] |
| 90 min tMCAO in SD rats | EA | LU5, LI4, ST36, SP6 | Started at 6 h post-modeling, 5 Hz dilatational waves, 2 mA, 20 min, once daily for 7 d | Ischemic penumbra: STAT6 mRNA and protein↑, p-STAT6↑, p-STAT6/STAT6↑, PPARγ mRNA and protein↑, p-PPARγ↑, NF-κB mRNA↓, p-NF-κB p65↓, TNF-α mRNA and protein↓, IL-6 mRNA and protein↓, TGF-β mRNA and protein↑, IL-10 mRNA and protein↑, CD86^+^ Iba1^+^ cells↓, CD206^+^ Iba1^+^ cells↑ | Microglia | Acute phase | [206] |
| 120 min tMCAO in SD rats | EA | GV20, LI4, LR3 | Started post-modeling, sequential stimulation of 20 Hz for 5 min then 2 Hz for 30 min, 1 mA, once daily for 3 d | Ischemic penumbra: A20 mRNA and protein↑, p-IKKβ/IKKβ↓, p-IκBα/IκBα↓, nuclear/cytoplasm NF-κB p65↓, TNF-α↓, IL-1β↓ | Microglia | Hyperacute phase | [210] |
| 120 min tMCAO in SD rats | EA | GV20, LI4, LR3 | Started post-modeling, sequential stimulation of 20 Hz for 5 min then 2 Hz for 30 min, 1 mA, once daily for 3 d | Ischemic penumbra: ABIN1 mRNA and protein↑, p-IκBα/IκBα↓, TNF-α↓, IL-1β↓, CCL2↓ | Microglia | Acute phase | [211] |
| 120 min tMCAO in SD rats | EA | GV20, LI4, LR3 | Started at 2 h post-modeling, sequential stimulation of 20 Hz for 5 min then 2 Hz for 30 min, 1 mA, once daily for 3 d | Ischemic penumbra: Iba1↓, CYLD mRNA and protein↑, p-IκBα/IκBα↓, nucleus/cytoplasm NF-κB p65↓, Bcl2a1a mRNA↓, TNF-α↓, IL-1β↓, CYLD^+^ NeuN^+^ cells↑, CX3CL1^+^ NeuN^+^ cells↓, CYLD^+^ NF-κB p65^+^ cells↑ | Microglia | Hyperacute and acute phase | [212] |
| 120 min tMCAO in SD rats | EA | GV20, LI4, LR3 | Started at 2 h post-modeling, sequential stimulation of 20 Hz for 5 min then 2 Hz for 30 min, 1 mA, once daily for 3 d | Ischemic penumbra cortex: CX3CL1↑, CX3CR1↓, NLRP3↓, Arg1 mRNA↑, Ym1 mRNA↑, Fizz1 mRNA↑, TNF-α mRNA↓, IL-1β mRNA↓, iNOS mRNA↓, CX3CL1^+^ CX3CR1^+^ cells↑, CD206^+^ Iba1^+^ cells↑, iNOS^+^ Iba1^+^ cells↓ | Microglia | Acute phase | [213] |
| 120 min tMCAO in SD rats | EA | LI11, ST36 | Started at 1 d post-modeling, 1/20 Hz dense-disperse waves, 4 V, 30 min, once daily for 3 d | Cortex: miR-9↑, IκBα↑, nucleus/cytoplasm NF-κB p65↓, TNF-α↓, IL-1β↓ | Microglia | Acute phase | [214] |
| 90 min tMCAO in SD rats | EA | GV20, GV24 | Started at 1 d post-modeling, 2/20 Hz dilatational waves, 6 V, 0.2 mA, 30 min, once daily for 7 d | CA1 and sensorimotor cortex: CD68↓, GFAP↓, IL-1β↓, IL-10↑, P2X7R^+^ CD68^+^ cells↓, P2X7R^+^ GFAP^+^ cells↓, P2Y1R^+^ CD68^+^ cells↓, P2Y1R^+^ GFAP^+^ cells↓ | Microglia | Acute phase | [217] |
| 90 min tMCAO in SD rats | EA | GV20, GV24 | Started at 1 d post-modeling, 2/20 Hz dilatational waves, 6 V, 0.2 mA, 30 min, once daily for 7 d | HIP: P2X7R↓, Nrf2↑, NLRP3↓, ROS↓, MDA↓, SOD↑, IL-1β↓, IL-6↓, iNOS↓, Arg1↑, IL-4↑, IL-10↑; DG: P2X7R^+^ Iba1^+^ cells↓, iNOS^+^ Iba1^+^ cells↓, Arg1^+^ Iba1^+^ cells↑ | Microglia | Acute phase | [218] |
| 120 min tMCAO in SD rats | EA | GV20, GV14, GV24 | Started at 2 h post-modeling, 2/5 Hz, 2 mA, 40 min, every 12 h for 7 treatments | Cortex: P2X7R mRNA and protein↓, NLRP3 mRNA and protein↓, ASC↓, Caspase-1↓, GSDMD mRNA and protein↓, IL-1β↓, IL-18↓ | Microglia | Acute phase | [219] |
| 120 min tMCAO in SD rats | EA | GV20, GV24 | Started at 1 d post-modeling, 1-20 Hz sparse-dense waves, 6 V, 30 min, once daily for 14 d | HIP: CD16↓, CD206↑, Nrf2 mRNA and protein↑, HO-1 mRNA and protein↑, TNF-α mRNA↓, IL-1β mRNA↓, IL-4 mRNA↑, IL-10 mRNA↑; Ischemic hemisphere: CD16^+^ Iba1^+^ cells↓, CD206^+^ Iba1^+^ cells↑; Serum: TNF-α↓, IL-1β↓, IL-4↑, IL-10↑ | Microglia | Subacute phase | [220] |
| 90 min tMCAO in SD rats | EP | GV20, GV14 | 3 Hz, 1-2 V, 30 min, once daily for 7 d | Ischemic penumbra: Iba1↓, CD206↑, RhoA↑, pyrin↓, GSDMD↓, Caspase-1↓, cleaved Caspase-1↓, ROCK2↓, RhoA^+^ Iba1^+^ cells↑, GSDMD^+^ Iba1^+^ cells↓ | Microglia | Hyperacute phase | [221] |
| 60 min tMCAO in C57BL/6 mice | EA | GV20, ST36 | Started at 1 d post-modeling, 2/15 Hz dispersive waves, 1 mA, 20 min, once daily for 5 d | HIP: NeuN mRNA and protein↑, Iba1 mRNA and protein↓, CD206 mRNA and protein↑, Abca1 mRNA and protein↑, CD206^+^ Iba1^+^ cells↑, Abca1^+^ NeuN^+^ CD206^+^ cells↑ | Microglia | Acute phase | [222] |
| pMCAO in Wistar rats | EA | GV20, GV14 | Started at 2 h post-modeling, 20 Hz, 1-2 mA, 30 min, once daily for 28 d | Ischemic penumbra: GFAP↑ | Astrocyte | Acute and subacute phase | [226] |
| pMCAO in SD rats | EA | LI11, ST36 | 15, 30 or 100 Hz dense-sparse waves, 60-80 μA, 20 min, once daily for 5 d | Brain tissues: GFAP↑ | Astrocyte | Acute phase | [227] |
| pMCAO in Wistar rats | EA | PC6 or SP8 | 2/15 Hz sparse-dense waves, 1 mA, 30 min, once daily for 14 d | HIP: GFAP mRNA and protein↑ | Astrocyte | Subacute phase | [228] |
| 120 min tMCAO in SD rats | EA | GV20, GV29, ST36 | Started on the day of modeling, 4/20 Hz dilatational waves, 10 min, once daily for 7 d | Ischemic penumbra: GFAP↑, PI3K↑, Akt↑, p-Akt↑; Serum: GFAP↑ | Astrocyte | Acute phase | [229] |
| 15 min tMCAO in SD rats | EA | GV20, GV14 | Started post-modeling, 4/20 Hz, 2-3 V, 1-3 mA, 30 min, once daily for 21 d | Ischemic penumbra: GFAP↓, S100B↓, p-p38↓, nuclear NF-κB p50↓, NF-κB p50↓, TNF-α↓, iNOS↓, TRADD↓, FADD↓, cleaved Caspase-8↓, cleaved Caspase-3↓, GFAP^+^ S100B^+^ cells↓, S100B^+^ RAGE^+^ cells↓, S100B^+^ nitrotyrosine^+^ cells↓ | Astrocyte | Acute phase | [230] |
| tMCAO in C57BL/6 mice | EA | GV20, ST36 | Started post-modeling, 2 Hz, 1 mA, 30 min, once daily for 3 d | Brain tissues: GFAP↓, Iba1↓, IL-33↓, ST2↓, resting Iba1^+^ cells↑, activated Iba1^+^ cells↓; Splenocytes and peripheral blood: CD4^+^ IFN-γ^+^ cells↓, CD4^+^ IL-17^+^IL-33^+^ cells↓ | Astrocyte | Acute phase | [232] |
| pMCAO in Wistar rats | EA | PC6, LI11 | Started at 2 h post-modeling, 2/15 Hz sparse-dense waves, 1 mA, 20 min, once daily for 7 d | Ischemic tissues: GFAP mRNA and protein↑, MCT1 mRNA and protein↑, Lactate↑ | Astrocyte | Acute phase | [234] |
| 120 min tMCAO in SD rats | EP | GV20 | 2/15 Hz, 1 mA, 30 min, once daily for 5 d, with modeling conducted at 24 h post-treatment | Brain tissues: NDRG2 mRNA and protein↓, nuclear NDRG2↓, NDRG2^+^ GFAP^+^ cells↓ | Astrocyte | Hyperacute phase | [235] |
| pMCAO in SD rats | EP | GV20, PC6, SP6 | 2/10 Hz dilatational waves, 6 V, 2 mA, 20 min, with modeling conducted at 24 h post-treatment | Ischemic penumbra cortex: GFAP↓, CD38↑, Ca2+↑, F-actin↑, Miro1↑, TRAK1↑, KIF5b↑, NeuN^+^ Mito-tracker^+^ cells↑ | Astrocyte | Hyperacute phase | [236] |
| 120 min tMCAO in SD rats | EA | GV20, ST36 | Started at 2 h post-modeling, 2 Hz continuous waves, 1 mA, 20 min, once daily for 2 d | Ischemic penumbra and core zone: MMP-2 mRNA and protein↓, AQP4 mRNA and protein↓, AQP9 mRNA and protein↓ | Neutrophil | Hyperacute phase | [241] |
|  | MA |  | Started at 2 h post-modeling, 1 min twisting (180°, 100±5 twists/min) at 10 min intervals, 20 min/session, once daily for 2 d | Ischemic penumbra and core zone: NeuN↑, MMP-2 mRNA and protein↓, AQP4 mRNA and protein↓, AQP9 mRNA and protein↓, MPO↓, CD68↓ |  |  |  |
| 120 min tMCAO in SD rats | EA | GV20, GV24 | Started at 2 h post-modeling, 1 and 2 Hz, 1-3 mA, 30 min, once daily for 7 d | HIP: MMP-2↓, MMP-9↓ | Neutrophil | Acute phase | [243] |
| 120 min tMCAO in SD rats | EA | GV20, LI4, LR3 | Started during MCAO (first session) and continued once daily for 6 d, 2 Hz, 1 mA, 30 min | Ischemic tissues: Bcl-2 mRNA and protein↑, Bax mRNA and protein↓, MMP-9 mRNA and protein↓, TIMP-1 mRNA and protein↑, BrdU^+^ GFAP^+^ cells↑↓, BrdU^+^ Nestin^+^ cells↑ | Neutrophil | Acute phase | [244] |
| 120 min tMCAO in SD rats | EA | GV20 | Started post-modeling, 2/15 Hz, 1 mA, 30 min, once daily for 2 d | Ischemic tissues: MMP-9 mRNA and protein↓, TIMP-2 mRNA and protein↑, H3K9ac/H3K27ac at MMP-9 promoter↓, H3K9ac/H3K27ac at TIMP-2 promoter↑ | Neutrophil | Hyperacute phase | [245] |
| 90 min tMCAO in C57BL/6 mice | EP | GV20 | 2/15 Hz, 1mA, 30 min, once daily for 2 d, with modeling conducted at 24 h post-treatment | Cortex: MCPIP1 mRNA and protein↑, MCPIP1^+^ NSE^+^ cells↑, TNF-α mRNA↓, IL-1β mRNA↓, IL-6 mRNA↓, CCL2 mRNA↓, p-NF-κB p65↓, leukocyte infiltration↓ | Monocyte | Hyperacute and acute phase | [249] |
| 30 min tMCAO in C57BL/6 mice | EA | GV20, GV26 | Started post-modeling, 4/20 Hz sparse-dense waves, 1-3 V, 1-3 mA, once daily for 4 d | Ischemic cortex: p-STAT3/STAT3↓, CCL2 mRNA↓, GzmB mRNA↓, NKG2D+ NK cells↓,IFN-γ+ NK cells↓, NK cells infiltration↓ | NK cell | Acute phase | [252] |
| 120 min tMCAO in SD rats | EP | GV20 | 2/15 Hz, 1 mA, 30 min, once daily for 5 d, with modeling conducted at 1 d post-treatment | Ischemic penumbra: α7nAChR↑, HMGB1↓; Plasma: HMGB1↓ | Cholinergic anti-inflammatory pathway | Acute phase | [260] |
| 90 min tMCAO in SD rats | EP | GV20 | 2/15 Hz, 1 mA, 30 min, once daily for 5 d, with modeling conducted at 1 d post-treatment | Ischemic penumbra: α7nAChR↑, NLRP3↓, pro-Caspase-1↓, Caspase-1↓, GSDMD↓, GSDMD-N↓, pro-IL-1β↓, IL-1β↓, IL-18↓, TNF-α↓, TGF-β1↑, IL-10↑ | Cholinergic anti-inflammatory pathway | Acute phase | [261] |
| 120 min tMCAO in SD rats | EA | GV20, GV24 | Started at 2 d post-modeling, 2-10 Hz dense-disperse waves, 2-4 mA, 30 min, once daily for 7 d | CA1: α7nAChR↑, Iba1↓, GFAP↓; HIP: TNF-α↓, IL-1β↓ | Cholinergic anti-inflammatory pathway | Subacute phase | [262] |
| 90 min tMCAO in SD rats | EP | GV20 | 2/15 Hz dense-disperse waves, 1 mA, 30 min, once daily for 5 d, with modeling conducted at 1 d post-treatment | Ischemic penumbra: α7nAChR↑, CD86↓, TNF-α↓, IL-1β↓, IL-6↓, iNOS↓, CD206↑, Arg1↑, TGF-β1↑, IL-4↑, IL-10↑, iNOS^+^ Iba1^+^ cells↓, Arg1^+^ Iba1^+^ cells↑ | Cholinergic anti-inflammatory pathway | Acute phase | [263] |
| 90 min tMCAO in SD rats | EA | GV20, GV14 | Started at 1 h post-modeling, 2/15 Hz sparse-dense waves, 1 mA, 30 min | Ischemic penumbra: ChAT mRNA↑, α7nAChR mRNA and protein↑, mAChR M1-5 mRNA↑, TNF-α↓, IL-10↑, MDA↓; Serum: TNF-α↓, IL-10↑; DMV: c-fos↑ | Cholinergic anti-inflammatory pathway | Hyperacute and acute phase | [264] |
| pMCAO in SD rats | EA | ST36 | Started post-modeling, 10 Hz continuous waves, 1 mA, 20 min, once daily for 7 d | Cortex and DMV: ChAT↑; Stomach: ChAT↑, α7nAChR↑; Serum: MTL↓, VIP↑, IL-6↓, TNF-α↓ | Cholinergic anti-inflammatory pathway | Acute phase | [265] |
| pMCAO in SD rats | LA | GV20, HT7 | Started at 2 d post-modeling, 5 min, every 2 d for 2 weeks | CA1: ChAT↑, CREB mRNA↑, BDNF mRNA↑, Bcl-2 mRNA↑, Bax mRNA↓ | Cholinergic anti-inflammatory pathway | Chronic phase | [266] |
| 60 min tMCAO in C57BL/6 mice and eNOS KO mice | EA | GV20, GV14 | Started at 20 min post-modeling, 2 Hz, 1 mA, 20 min | Brain tissues: ACh↑, mAChR M3↑ | Cholinergic anti-inflammatory pathway | Hyperacute phase | [267] |
| pMCAO in SD rats | EA | GV20, GV26 | Started at 1 h post-modeling, 2 Hz, 1 mA, 20 min | Leptomeningeal collateral diameter↑, vascular resistance↓, nucleus basalis of meynert cholinergic neuron activity↑ (discharge rates↑, c-Fos↑, ChAT↑) | Cholinergic anti-inflammatory pathway | Hyperacute phase | [268] |
| 120 min tMCAO in SD rats | EP | GV20, ST36 | Started at 2 d post-modeling, 2/15 Hz dense-sparse waves, 1 mA, 30 min, once daily with 6 sessions/week for 14 d | Ileum: Iba1↓, IL-1β↓, IFN-γ↓, TGF-β1↑, IL-4↑, IL-18↓, TLR4↓, NF-κB p65↓, NLRP3↓, Caspase-1 p20↓, TRX1↑, TXNIP↓, ZO-1↑, Occludin↑; Plasma: LPS↓, DAO↓, D-lactic acid↓ | Brain-gut axis | Acute phase | [269] |
| 120 min tMCAO in SD rats | EA | GV20 | Started post-modeling, 2/15 Hz, 1 mA, 20 min, once daily for 2 d | Ischemic tissues: TNF-α mRNA↓, IL-1β mRNA↓; Small intestine: TNF-α mRNA and protein↓, IL-1β mRNA and protein↓, CXCL1 mRNA↓, CXCL2 mRNA↓, IL-10↑, ZO-1↑, Occludin↑, Claudin-1↑, Treg/γδ T cells↑; Serum: TNF-α↓, IL-1β↓, IL-10↑, DAO↓, D-lactate acid↓ | Brain-gut axis | Hyperacute phase | [270] |
| 120 min tMCAO in SD rats | EP | GV20, GB7 | 2-20 Hz, 30min, once daily for 3 d | Brain tissues: NF-κB p65 mRNA and protein↓, NLRP3 mRNA and protein↓, Caspase-3 mRNA and protein↓, Caspase-9 mRNA and protein↓; Serum: TNF-α↓, IL-6↓, TMAO↓, IL-4↑; Intestine: n-propyl acetate↑ | Brain-gut axis | Hyperacute phase | [274] |
| 90 min tMCAO in C57BL/6 mice | EA | GV20, GV14, ST36 | Started for 5 d before modeling, 2-15 Hz, 2 mA, modeling conducted at 24 h after the final session | Gut: Akkermansia↑, Lactobacillus↑, Escherichia shigella↓, IPA↑; Ischemia penumbra: MT1↑, PGC-1α↑, UCP2↑, cleaved Caspase-3↓, Bcl-2/Bax↑, TAOC↑, SOD↑, GSH/GSSG↑, MDA↓ | Brain-gut axis | Hyperacute phase | [276] |
| 120 min tMCAO in SD rats | EA | GV20 | Started at 1 d post-modeling, 2/15 Hz, 1 mA, 30 min, once daily for 4 d | Ischemic tissues: TNF-α mRNA and protein↓, IL-1β mRNA and protein↓, IL-10 mRNA and protein↑, Foxp3^+^/CD45^+^ CD4^+^ cells↑; Small intestine：TNF-α mRNA↓, IL-1β mRNA↓, IL-10 mRNA↑, Foxp3^+^/CD45^+^ CD4^+^ cells↑, CD4^+^ cells↓, acetate↑, propionate↑, butyrate↑, p-Bacteroidota↑, p-Firmicutes↑, f-Muriaculaceae↑, f-Lachnospiraceae↑, f-Ruminococcaceae↑; colonic contents: acetate↑, propionate↑, butyrate↑; intestine：Foxp3 mRNA and protein↑, acetylated-Foxp3↑ | Brain-gut axis | Acute phase | [277] |
| 90 min tMCAO in SD rats | EA | LI11, ST36 | Started at 1 d post-modeling, 1/20 Hz dilatational waves, 6 V, 30min, once daily for 14 d | Peripheral blood: total SCFAs↑, acetate↑, propionate↑ | Brain-gut axis | Chronic phase | [278] |
| 120 min tMCAO in SD rats | EA | GV20 | Started post-modeling, 2/15 Hz, 1 mA, 20 min, once daily for 4 d | Ischemic tissues and small intestine: TNF-α↓, IL-1β↓, IL-17↓, IL-10↑, CXCL1 mRNA↓, CXCL2 mRNA↓, Foxp3^+^ cells↑, TCR γδ^+^ cells↓, Treg/γδ T cells↑, CM-Dil^+^ CD3^+^ cells↓, CM-Dil^+^ TCR γδ^+^ cells↓; Ischemic tissues: CD3^+^ TCRγδ^+^ CFSE^+^ cells↓; Small intestine: CD45^+^ TCRγδ^+^ CFSE^+^ cells↓ | Brain-gut axis | Acute phase | [279] |

***Notes:*** ↑, upregulated by acupuncture; ↓, downregulated by acupuncture. **Abbreviation:** 2-AG, 2-arachidonoylglycerol; 5-HT, 5-hydroxytryptamine; 5-HT_2_A, 5-HT receptor 2A; A_1_R, adenosine A_1_ receptor; A20, TNF alpha-induced protein 3; AANAT, arylalkylamine N-acetyltransferase; ABIN1, A20-binding inhibitor of NF-κB activation 1; AC, adenylate cyclase; AcbSh, nucleus accumbens shell; ACh, acetylcholine; ACTH, adrenocorticotropic hormone; AEA, anandamide; Akt, protein kinase B; ALFF, amplitude of low-frequency fluctuation; AMPAR, α-amino-3-hydroxy-5-methyl-4-isoxazolepropionic acid receptor; Ang II, angiotensin II; ANXA1, annexin A1; AQP4, aquaporin 4; AQP9, aquaporin 9; Arg1, arginase 1; ASC, apoptosis-associated speck-like protein containing a CARD; AT_1_R, angiotensin II receptor type 1; AT_2_R, angiotensin II receptor type 2; AUD, auditory cortex; Bax, BCL2-associated X protein; BC, betweenness centrality; Bcl-2, B-cell lymphoma 2; BDNF, brain-derived neurotrophic factor; Bmal1, brain and muscle ARNT-like 1; BrdU, bromodeoxyuridine; CaM, calmodulin; CaMKII, Ca^2+^/calmodulin-dependent protein kinase II; cAMP, cyclic adenosine monophosphate; Caspase-1, cysteinyl aspartate-specific protease-1; Caspase-3, cysteinyl aspartate-specific protease-3; Caspase-8, cysteinyl aspartate-specific protease-8; CB_1_R, cannabinoid receptor type 1; CC, corpus callosum; CCL2, C-C motif chemokine ligand 2; CD, cluster of differentiation; CDK, cyclin-dependent kinase; CFSE, carboxyfluorescein succinimidyl ester; CG, cingulate gyrus; ChAT, choline acetyltransferase; Clock, circadian locomotor output cycles kaput; CM-Dil, chloromethyl-1,1'-dioctadecyl-3,3,3',3'-tetramethylindocarbocyanine perchlorate; COX IV, cytochrome c oxidase subunit 4; CREB, cAMP-response element binding protein; CX3CL1, C-X3-C motif chemokine ligand 1; CX3CR1, C-X3-C motif chemokine receptor 1; CXCL2, C-X-C motif chemokine ligand 2; Cx43, connexin 43; cyto c, cytochrome c; CYLD, cylindromatosis; c-Fos, proto-oncogene c-Fos; DAG, diacylglycerol; DAO, diamine oxidase; DC, degree centrality; DCX, doublecortin; DG, dentate gyrus; DMV, dorsal motor nucleus of the vagus; DT, dorsal thalamus; EA, electroacupuncture; EdU, 5-ethynyl-2'-deoxyuridine; EMG, electromyography; eNOS, endothelial nitric oxide synthase; EP, electroacupuncture pretreatment; EPO, erythropoietin; EpoR, erythropoietin receptor; ER, estrogen receptor; ERK1/2, extracellular signal-regulated kinase 1/2; F-actin, filamentous actin; FADD, Fas-associated protein with death domain; FC, functional connectivity; Fizz1, found in inflammatory zone 1; FNDC5, fibronectin type III domain-containing protein 5; Foxp3, forkhead box P3; FPR, formyl peptide receptor; GABA, gamma-aminobutyric acid; GABA_A_R, GABA type A receptor; GABA_A_Rγ2, GABA type A receptor gamma 2 subunit; GABA_B_1, GABA type B receptor subunit 1; GABA_B_2, GABA type B receptor subunit 2; GABAT, GABA transaminase; GAP-43, growth-associated protein 43; Gas7, growth arrest-specific 7; GDNF, glial cell line-derived neurotrophic factor; GFAP, glial fibrillary acidic protein; GLT-1, Glu transporter 1; Glu, glutamate; GluN1, glutamate ionotropic receptor NMDA type subunit 1; GluN2A, glutamate ionotropic receptor NMDA type subunit 2A; GluN2B, glutamate ionotropic receptor NMDA type subunit 2B; G_q_, Gq protein; Grm1a, glutamate metabotropic receptor 1a; GSDMD, gasdermin D; GSH, glutathione; GSK-3, glycogen synthase kinase-3; GSK-3β, glycogen synthase kinase-3β; GSSG, oxidized glutathione; GzmB, granzyme B; Hes1, hairy and enhancer of split 1; HIF-1α, hypoxia-inducible factor 1-alpha; HIP, hippocampus; HMGB1, high mobility group box 1; HO-1, heme oxygenase 1; HSP70, heat shock protein 70; H3K27ac, histone H3 lysine 27 acetylation; H3K9ac, histone H3 lysine 9 acetylation; Iba1, ionized calcium-binding adapter molecule 1; IFN-γ, interferon-gamma; IKK, IκB kinase; IL, interleukin; iNOS, inducible nitric oxide synthase; IP_3_, inositol trisphosphate; IPA, indole-3-propionic acid; IκBα, inhibitor of kappa B alpha; JAK2, Janus kinase 2; JNK, c-Jun N-terminal kinase; KCC2, K⁺-Cl⁻ cotransporter 2; KIF5b, kinesin family member 5B; L1cam, L1 cell adhesion molecule; LA, laser acupuncture; LC3II/I, microtubule-associated protein 1A/1B-light chain 3; LIMK1, LIM domain kinase 1; LINGO-1, leucine-rich repeat and immunoglobulin domain-containing protein 1; LncRNA, long non-coding RNA; LPA, lysophosphatidic acid; LPS, lipopolysaccharide; M1, primary motor cortex; MA, manual acupuncture; MAP2, microtubule-associated protein 2; MBP, myelin basic protein; MC, motor cortex; m-calpain, muscle calpain; MCPIP1, monocyte chemotactic protein-induced protein 1; MCT1, monocarboxylate transporter 1; MDA, malondialdehyde; Met-enkephalin, methionine-enkephalin; mAChR, muscarinic acetylcholine receptor; MLC1, myosin light chain 1; MMP, matrix metalloproteinas; MPO, myeloperoxidase; mPTP, mitochondrial permeability transition pore; miR, microRNA; Miro1, mitochondrial Rho GTPase 1; MT, melatonin receptor; mtDNA, mitochondrial DNA; MTL, motilin; mTOR, mechanistic target of rapamycin; MyD88, myeloid differentiation primary response 88; MYPT1, myosin phosphatase target subunit 1; NDRG2, n-myc downstream regulated gene 2; NeuN, neuronal nuclei; NeuroD1, neurogenic differentiation factor 1; NF-κB, nuclear factor kappa B; NGF, nerve growth factor; NGL-3, netrin-G ligand-3; NgR, Nogo receptor; NK cells, natural killer cells; NKG2D, natural killer group 2D; NLRP3, NOD-like receptor family pyrin domain containing 3; Nogo-A, neurite outgrowth inhibitor A; Notch, neurogenic locus notch homolog protein; NRF1, nuclear respiratory factor 1; Nrf2, nuclear factor erythroid 2-related factor 2; NSE, neuron-specific enolase; OMgp, oligodendrocyte-myelin glycoprotein; OTULIN, OTU deubiquitinase with linear linkage specificity; p21, cyclin-dependent kinase inhibitor 1A; p27, cyclin-dependent kinase inhibitor 1B; p38, p38 mitogen-activated protein kinase; p75^NTR^, p75 neurotrophin receptor; P2X7R, purinergic receptor P2X7; P2Y1R, purinergic receptor P2Y1; Parkin, Parkin RBR E3 ubiquitin protein ligase; PCNA, proliferating cell nuclear antigen; PFC, prefrontal cortex; PGC-1α, peroxisome proliferator-activated receptor gamma coactivator 1-alpha; PI3K, phosphatidylinositol 3-kinase; PINK1, PTEN-induced putative kinase 1; PIR, piriform cortex; PirB, paired immunoglobulin-like receptor B; PKA, protein kinase A; PKCε, protein kinase C epsilon; pMCAO, Permanent Middle Cerebral Artery Occlusion; PPARγ, peroxisome proliferator-activated receptor gamma; PRG5, plasticity-related gene 5; pro-BDNF, precursor of brain-derived neurotrophic factor; PSD-95, postsynaptic density protein 95; PTEN, phosphatase and tensin homolog; Rac1, Ras-related C3 botulinum toxin substrate 1; RAGE, receptor for advanced glycation end products; Rb, retinoblastoma protein; ReHo, regional homogeneity; RhoA, Ras homolog family member A; ROCK2, Rho-associated coiled-coil containing protein kinase 2; ROS, reactive oxygen species; RSP, retrosplenial cortex; S1, primary somatosensory cortex; S100B, S100 calcium-binding protein B; S6, ribosomal protein S6; SCFAs, short-chain fatty acids; SERT, serotonin transporter; SOD, superoxide dismutase; Sox2, SRY-box transcription factor 2; SP, substance P; Src, Src tyrosine kinase; SSC, somatosensory cortex; STAT3, signal transducer and activator of transcription 3; STAT5, signal transducer and activator of transcription 5; ST2, growth stimulation-expressed gene 2; STR, striatum; SUMO, small ubiquitin-like modifier; SVZ, subventricular zone; SYN, synaptophysin; sEPSCs, spontaneous excitatory postsynaptic currents; TAOC, total antioxidant capacity; TAZ, tafazzin; TCR, T cell receptor; TFAM, mitochondrial transcription factor A; TGF-β, transforming growth factor-beta; Th, T helper cells; TIMP, tissue inhibitor of metalloproteinase; TLR4, Toll-like receptor 4; TMAO, trimethylamine N-oxide; tMCAO, transient middle cerebral artery occlusion; TNF-α, tumor necrosis factor-alpha; tPA, tissue-type plasminogen activator; TRADD, TNFRSF1A-associated via death domain; TRAF6, TNF receptor associated factor 6; TRAK1, trafficking kinesin protein 1; TREM2, triggering receptor expressed on myeloid cells 2; TrkA, tropomyosin receptor kinase A; TrkB, tropomyosin receptor kinase B; RPV1/4, transient receptor potential vanilloid 1/4; TRX1, thioredoxin 1; TXNIP, thioredoxin interacting protein; UCP2, uncoupling protein 2; VEGF, vascular endothelial growth factor; VGLUT1, vesicular glutamate transporter 1; VIP, vasoactive intestinal peptide; VIS, visual cortex; Wnt1, Wnt family member 1; YAP, Yes1 associated transcriptional regulator; Ym1, chitinase 3 like 3; ZO-1, zonula occludens-1; α7nAChR, α7 nicotinic acetylcholine receptor; γδ T cells, gamma delta T cells.
